# Beyond personality traits: a motivation–self-regulation model of mathematical problem-solving through self-efficacy and mathematical thinking

**DOI:** 10.3389/fpsyg.2026.1803867

**Published:** 2026-04-13

**Authors:** Jie Wang, Lin Guo

**Affiliations:** Faculty of Education, Languages, Psychology & Music, SEGi University Kota Damansara, Petaling Jaya, Malaysia

**Keywords:** Big Five framework, high school, learning motivation, math education, mathematical thinking

## Abstract

**Purpose:**

This study aims to understand how learning-related traits and process variables collectively influence high school students’ mathematical problem-solving ability; and to verify the mediating roles of mathematical self-efficacy and mathematical thinking.

**Methods:**

Based on questionnaire and mathematical performance test data from 1,183 high school students in Shanghai, the study employs partial least squares structural equation modeling (PLS-SEM) for analysis.

**Results:**

The results show that the model has high explanatory power for mathematical self-efficacy, mathematical thinking, and mathematical problem-solving ability; motivation to math and self-regulated learning are core upstream predictors of problem-solving performance, while mathematical self-efficacy and mathematical thinking serve as the closest direct predictors of problem-solving and significantly mediate the effects of motivation and self-regulated learning on problem-solving ability. In contrast, personality traits exhibit relatively weak direct and mediating effects after controlling for prior academic performance and the aforementioned learning variables.

**Implications:**

The contribution of this study lies not only in integrating motivation, self-regulation, affective beliefs, and higher-order mathematical thinking into a unified structural framework, but also in clarifying the relative explanatory roles of distal personality traits and proximal learning processes, showing that mathematical problem-solving is shaped through both affective-belief and cognitive pathways, and demonstrating that the explanatory advantage within the present model lies primarily in domain-specific, modifiable learning processes rather than broad personality dispositions.

## 1 Introduction

The rapid development of social and digital economies in the 21st century has made the ability to “use mathematics to solve complex real-world problems” a core objective in the reform of basic education and the cultivation of talent in higher education across nations. Whether in national curriculum standards or large-scale assessments, there is an ongoing emphasis on moving beyond rote computation in mathematics learning, shifting toward higher-level mathematical thinking activities such as modeling, reasoning, argumentation, and reflection (Horrocks and Shearman, 2025; Kappassova et al., 2025). Meanwhile, the widespread adoption of artificial intelligence and data technologies has exposed students to increasingly open-ended, interdisciplinary contextual problems in their studies and daily lives, making standard textbook problem types insufficient to address these challenges (George, 2023). This reality has prompted researchers and frontline educators to gradually shift focus from “whether students can solve problems” to examining “why students are willing to learn mathematics, how they regulate their own learning behaviors, whether they believe they can master mathematics, and the systematic relationships between these internal psychological factors and mathematical thinking, problem-solving” (Lin, 2023; Menlah and Boateng, 2025).

Extensive classroom observations and assessment results indicate that many students grasp basic formulas and problem-solving steps but struggle when faced with slightly modified or modeling-involving problems, manifesting as reluctance to attempt, lack of persistence, and rigid reliance on templates without flexible adaptation (Prabawanto et al., 2024; Worku et al., 2025). Even among students with comparable knowledge levels, there remains significant variation in mathematical problem-solving performance, which cannot be fully explained by cognitive abilities or classroom instruction alone (Stenberg Hartviksen and Haavold, 2025; Xu and Li, 2025). This disparity is closely linked to individual personality traits (e.g., conscientiousness, emotional stability), motivation in mathematics, self-regulated learning habits, and math self-efficacy (Cupani et al., 2025). However, in practical teaching, these factors are often addressed piecemeal rather than through a systematic model that integrates “who the student is” (personality traits), “why they are willing to learn and how they manage learning” (motivation to learn math and self-regulated learning), “whether they believe they can succeed in math” (math self-efficacy), and “how to engage in mathematical thinking and ultimately solve problems” (mathematical thinking and problem-solving skills). This study seeks to address this practical challenge by employing structural equation modeling to delineate the comprehensive mechanism from traits to problem-solving, providing theoretical foundations and empirical support for targeted interventions and instructional design in mathematics education.

Existing research has gradually shifted from solely focusing on students’ cognitive abilities to examining the roles of self-regulated learning, self-assessment, and metacognition in mathematical problem-solving. In the “Digital Math Training” project in Italy, Barana et al. (2022) compared the consistency between students’ self-evaluation of their problem-solving processes and instructor ratings through online training on eight real-world scenario problems. They found a significant positive correlation overall but noted that students consistently underestimated their performance. Additionally, students’ self-assessments were more accurate in dimensions such as “problem-solving process development” and “use of technical tools,” but exhibited larger discrepancies in higher-order mathematical thinking aspects like “contextual understanding” and “strategic justification.” This indicates that metacognitive monitoring and self-reflection surrounding problem-solving are not only critical components of mathematical problem-solving ability but also key entry points for cultivating students’ self-regulated learning capabilities. Fathi et al. (2023), based on Zimmerman (2023)‘s tripartite self-regulation theory, constructed a high-level self-regulation structural equation model encompassing motivational regulation (self-efficacy, task value, mastery goal orientation), cognitive regulation (elaboration, critical thinking), and behavioral regulation (self-observation, self-judgment, self-reaction) to examine their impact on 11th-grade students’ mathematical reasoning and academic performance. The results revealed that behavioral regulation was the “determining factor” influencing both reasoning ability and achievement, while motivational regulation was the dominant factor affecting mathematical reasoning. Cognitive regulation played a significant mediating role between motivational regulation and reasoning, and together with behavioral regulation and reasoning, it mediated the influence of motivation on academic performance. Yu (2023) approached the issue from another angle by breaking down self-regulated learning into three dimensions: task behavior, motivation (intrinsic/extrinsic), and metacognition (online/offline). These studies collectively demonstrate that, whether approached through self-assessment, behavioral regulation, or metacognitive monitoring, students’ motivation, self-regulatory processes, and high-level mathematical thinking are closely interconnected and serve as critical antecedents for enhancing mathematical problem-solving abilities.

However, the gap in the literature is not simply that these variables have rarely appeared in the same statistical model. The deeper problem is that existing social-cognitive and expectancy-value perspectives have not clearly established the relative explanatory status of distal personality traits and proximal learning processes in higher-order mathematical problem-solving. Research on self-regulation has been dominated by process-based accounts, emphasizing variables such as self-evaluation, homework behavior, motivation regulation, and cognitive strategies (Afzaal et al., 2024; Begum, 2025). Although this work has clarified how students regulate learning in mathematics-related settings, it has seldom examined these process variables together with relatively stable personality dispositions within a single mathematics-specific framework. Consequently, it remains unclear whether broad dispositional tendencies retain independent explanatory value once domain-specific motivation, self-regulated learning, and prior achievement are taken into account, or whether their effects are largely absorbed by more proximal, malleable processes.

A second unresolved issue concerns mechanism. Prior studies have typically privileged either affective-belief processes, such as self-efficacy, or cognitive processes, such as reasoning, metacognition, and modeling, but have rarely treated mathematical self-efficacy and mathematical thinking as analytically distinct pathways linking learning processes to problem-solving performance. This distinction is theoretically consequential. Mathematical self-efficacy reflects students’ beliefs about their capability to engage successfully with mathematical tasks, whereas mathematical thinking refers to the quality of higher-order cognitive activity involved in representing, analyzing, and solving problems. Yet these levels are often conflated. Kryshko et al. (2022), for example, located self-efficacy within motivational regulation, whereas Arianto and Hanif (2024) treated metacognition as part of self-regulation in predicting problem-solving outcomes. Barana et al. (2022) focused on self-assessment accuracy across stages of problem solving, and Hemmler and Ifenthaler (2024) explicitly called for finer-grained distinctions among self-regulation processes across cultural and classroom contexts. Taken together, this literature suggests conceptual progress, but not conceptual resolution: it still does not clearly differentiate mathematical thinking quality from problem-solving performance, nor does it directly test whether mathematical self-efficacy and mathematical thinking transmit effects through separate mediating channels.

This ambiguity is especially consequential in a high-performance educational context such as Shanghai. Under conditions of strong instructional routines, examination pressure, and highly standardized performance norms, observable variation in students’ learning behavior may be compressed. In such settings, the apparent contribution of broad traits may weaken, while the explanatory salience of proximal learning processes may increase. For this reason, the present study is not intended as a broader aggregation of familiar predictors. Rather, it addresses three sharper theoretical questions: which class of predictors retains greater explanatory priority when traits and processes are modeled simultaneously; whether mathematical self-efficacy and mathematical thinking operate as distinct mediating mechanisms; and how a high-performance educational system may reshape the relative visibility of trait-based and process-based influences. Framed this way, the study seeks not merely to test direct and indirect paths, but to clarify the architecture of explanation in mathematical problem-solving by specifying how distal dispositions, proximal learning processes, affective-belief mechanisms, and higher-order mathematical thinking jointly relate to performance. Therefore, this study aims to answer:

*RQ1*: How do personality traits, motivation to math, self-regulated learning, and math self-efficacy influence students’ mathematical problem-solving skills?

*RQ2*: What are the roles of mathematical thinking and math self-efficacy play in the model?

## 2 Literature review

This review is organized around a layered and critical explanatory logic rather than a simple accumulation of predictors. Specifically, it distinguishes between distal individual differences, represented by personality traits, and proximal, modifiable learning processes, represented by motivation to math and self-regulated learning. It further proposes that these antecedents may influence mathematical problem-solving through two parallel mechanisms: an affective-belief mechanism, reflected in math self-efficacy, and a cognitive mechanism, reflected in mathematical thinking. Framed in this way, the present model is intended not merely to integrate variables that have already been studied separately, but to clarify their relative explanatory roles within a mathematics-specific account of higher-order problem-solving. This perspective builds on existing work in personality research, social-cognitive theory, expectancy-value theory, and self-regulated learning, while addressing the limited attention that prior studies have paid to the hierarchical relationship between broad dispositions, malleable learning processes, and domain-specific mechanisms in mathematics achievement and problem-solving.

This layered perspective also responds to a broader tension in the recent literature. On the one hand, research in mathematics education and adjacent educational psychology has increasingly emphasized self-regulated learning, motivational beliefs, metacognition, and higher-order reasoning as central to students’ performance on non-routine and cognitively demanding tasks. On the other hand, broader meta-analytic and longitudinal evidence continues to show that Big Five personality traits, especially conscientiousness are associated with academic achievement, although such effects are often modest and may depend on whether more proximal learning processes are included in the model. By placing these two lines of research into direct dialog, the present study moves beyond a variable-by-variable review and instead asks a more theoretically consequential question: when broad traits, domain-specific motivation, self-regulated learning, self-efficacy, and mathematical thinking are considered together, which set of factors remains most explanatory for higher-order mathematical problem-solving?

Taken together, the reviewed literature suggests that the key issue is not whether personality traits, motivation, self-regulated learning, self-efficacy, and mathematical thinking are each individually relevant, because prior studies already indicate that they are. The more important unresolved issue is how these predictors should be theoretically ordered. The present study argues that broad personality traits are better understood as distal conditions, whereas motivation to math and self-regulated learning function as more proximal and modifiable learning processes. These processes are then expected to operate through two mathematics-specific mechanisms: an affective-belief pathway, reflected in mathematical self-efficacy, and a cognitive pathway, reflected in mathematical thinking. Such a framework allows the study to move beyond citation accumulation and toward a more critically integrated account of higher-order mathematical problem-solving, in which the central question is not simply which variables matter, but how their explanatory roles differ in level, mechanism, and contextual relevance. This question is particularly important because mathematics education research has increasingly shifted from routine achievement outcomes toward reasoning, modeling, and problem-solving as focal indicators of students’ deeper learning, yet the theoretical hierarchy among trait-based, process-based, affective, and cognitive predictors remains insufficiently clarified.

Personality traits capture relatively stable patterns of thoughts, emotions, and behaviors that shape how students approach academic tasks (Bleidorn et al., 2021). In the Big Five framework, traits such as conscientiousness, emotional stability, and openness to experience are particularly relevant for learning in demanding domains like mathematics (Kibtiyah and Suud, 2024). Students who are more conscientious tend to be organized, persistent, and willing to invest effort, whereas emotionally stable students are less likely to experience debilitating anxiety in evaluative situations (Sarwer et al., 2025). Adaptive traits are positively associated with academic self-beliefs, including general academic self-efficacy and domain-specific confidence in mathematics, because they foster a history of successful experiences, effective coping, and persistence in the face of difficulty (Abood et al., 2020). At the same time, broader large-sample and meta-analytic research has generally shown that personality traits, especially conscientiousness, are positively associated with academic achievement, although the magnitude of these relationships is often modest and may vary depending on whether more proximal predictors are considered simultaneously (Ackerman and Kanfer, 2025; Mammadov, 2022). This suggests that the role of personality in mathematics learning should not be assumed to be either negligible or dominant, but instead examined in relation to domain-specific motivational and self-regulatory processes. When students perceive themselves as diligent, emotionally resilient, and capable of handling complex tasks, they are more likely to believe that they can master mathematical content and solve challenging problems (Shengyao et al., 2024). Therefore, we propose:

*H1a*: Personality traits positively predict students’ math self-efficacy.

Motivation to math reflects the extent to which students value learning mathematics, expect success in math-related tasks, and are willing to invest effort and persistence in the subject (Middleton and Spanias, 1999). From expectancy–value and self-determination perspectives, students who hold stronger intrinsic interest and higher task value in mathematics are more likely to engage deeply with learning activities, seek challenges, and interpret difficulties as opportunities for growth rather than as threats. Such motivated engagement tends to generate more frequent mastery experiences, constructive feedback, and a sense of progress, which are central sources of self-efficacy beliefs (Zhao and Ma, 2025). When students repeatedly experience that their invested effort in mathematics leads to understanding, improvement, and successful problem solving, they are more inclined to believe that they can handle future mathematical tasks (Bendol and Jr, 2025). Conversely, low motivation often results in superficial engagement and avoidance of challenging problems, limiting opportunities to build confidence (Kamberi, 2025). On this basis, it is reasonable to expect that students with higher motivation to learn mathematics will report stronger math self-efficacy (Zakariya et al., 2022). Therefore, we propose the following hypothesis:

*H1b*: Motivation to math positively predicts students’ math self-efficacy.

Self-regulated learning refers to students’ proactive use of goal setting, strategic planning, monitoring, and regulation of their own cognition, motivation, and behavior during learning (Brenner, 2022). Within social cognitive theory, self-regulated learners are not passive recipients of instruction; instead, they actively organize their study time, select appropriate strategies, seek help when needed, and reflect on their progress (Jartitngarm, 2025). In mathematics, such regulatory practices increase the likelihood that students will transform momentary confusion into eventual understanding, because they persist longer, try alternative approaches, and deliberately learn from errors (Mangarin and Caballes, 2024). These successful experiences, in turn, provide powerful mastery evidence for building self-efficacy beliefs. When students repeatedly observe that their deliberate regulation of effort and strategy leads to improved performance and clearer comprehension in mathematics, they are more likely to view themselves as capable of mastering future mathematical tasks (Aydan and Capa-Aydin, 2025). By contrast, students who rarely plan, monitor, or adjust their learning are more prone to experience failure as uncontrollable, which undermines their confidence. Thus, higher levels of self-regulated learning should be associated with stronger math self-efficacy. Hence, we posit:

*H1c*: Self-regulated learning positively predicts students’ math self-efficacy.

Mathematical thinking involves higher-order processes such as logical reasoning, generalization, modeling, and justification, which enable students to move beyond routine procedures and flexibly tackle unfamiliar problems (Genç et al., 2025). Personality traits provide a dispositional basis for how students approach such cognitively demanding tasks. In particular, conscientiousness is associated with persistence, carefulness, and thoroughness, which are essential for checking assumptions, exploring multiple solution paths, and refining arguments in mathematical reasoning (Dang et al., 2025). Openness to experience is linked to curiosity, preference for complexity, and willingness to explore novel ideas, all of which support the search for patterns, the construction of models, and the abstraction of underlying structures from concrete situations. Emotional stability helps students manage anxiety and frustration when grappling with challenging problems, allowing them to maintain cognitive resources for systematic thinking rather than resorting to avoidance or guesswork (Liu, 2025). When these adaptive traits are present, students are more likely to engage deeply with mathematical content, to reflect on their own reasoning, and to develop more sophisticated ways of thinking about mathematical relationships. Accordingly, students with more favorable personality profiles can be expected to display higher levels of mathematical thinking. Hence, we assume:

*H2a*: Personality traits positively predict students’ mathematical thinking.

Motivation to math shapes the depth and quality of students’ engagement with mathematical ideas. Students who perceive mathematics as valuable, interesting, and meaningful are more willing to invest time and cognitive effort in exploring concepts, making connections, and tackling non-routine problems (Supriadi et al., 2024). From expectancy–value and self-determination perspectives, high motivation leads learners to pursue understanding rather than mere task completion, to persist when initial solution attempts fail, and to voluntarily engage with challenging tasks that require reasoning, conjecturing, and justifying (Luria et al., 2021). Such sustained, effortful engagement is precisely the context in which mathematical thinking develops: students practice analyzing structures, comparing solution strategies, generalizing from specific examples, and constructing arguments to support their conclusions (Johansson and Sumpter, 2025). In contrast, students with low motivation are more likely to rely on rote procedures, avoid difficult items, and terminate exploration once a minimal answer is found, which restricts opportunities to cultivate higher-order mathematical thinking (Stavropoulou et al., 2025). Hence, stronger motivation to learn mathematics should be associated with more advanced and flexible mathematical thinking. Hence, we posit:

*H2b*: Motivation to math positively predicts students’ mathematical thinking.

Self-regulated learning provides a process-oriented foundation for the development of mathematical thinking. Students who set clear goals, plan their study activities, monitor their understanding, and adjust strategies when facing difficulty are more likely to move beyond mechanical application of procedures toward reflective and flexible reasoning (Zarestky et al., 2022). In mathematics, such learners actively check the plausibility of their solutions, compare alternative methods, and seek to understand why a particular strategy works, rather than merely whether it yields the correct answer (Smit et al., 2022). Their use of metacognitive regulation, such as evaluating progress, questioning underlying assumptions, and revising their approach directly supports key components of mathematical thinking, including analyzing structures, making generalizations, and constructing logical justifications (Zhang et al., 2024). Moreover, self-regulated learners tend to persist with non-routine problems and view errors as information for refining their reasoning, which creates rich opportunities to practice and internalize higher-order mathematical processes (Huang et al., 2024). Consequently, students who display stronger self-regulated learning behaviors are expected to exhibit higher levels of mathematical thinking. Hence, we propose:

*H2c*: Self-regulated learning positively predicts students’ mathematical thinking.

Math self-efficacy refers to students’ beliefs about their capability to successfully perform tasks in mathematics, such as understanding new concepts, solving challenging problems, or performing well on exams (Zuo et al., 2024). Within social cognitive theory, self-efficacy is a central determinant of how people choose tasks, how much effort they invest, how long they persist in the face of obstacles, and how resilient they are after failure (Jartitngarm, 2025). In the context of mathematics learning, students with higher math self-efficacy are more likely to attempt difficult problems, to persist when their first strategy does not work, and to mobilize a wider repertoire of solution methods (Velez and Abuzo, 2024). They also tend to interpret temporary setbacks as challenges that can be overcome through effort and strategy adjustment, rather than as evidence of inability. Such patterns of perseverance, strategic experimentation, and adaptive interpretation of feedback directly support more effective problem-solving performance. This mechanism is also consistent with evidence from other educational contexts. Self-efficacy significantly mediated the effects of digital competence and psychological well-being on students’ career choice, suggesting that self-efficacy functions as a meaningful transmission mechanism through which broader antecedent conditions are converted into consequential student outcomes in Indonesia (Kholifah et al., 2025). In contrast, students with low math self-efficacy are prone to avoidance, early withdrawal, and reliance on guessing or rote procedures, all of which undermine problem-solving success (Zakariya, 2022). Accordingly, stronger math self-efficacy should be associated with higher levels of mathematical problem-solving skills.

Mathematical problem-solving skills require more than the application of memorized procedures; they depend critically on the quality of students’ mathematical thinking (Suryawan et al., 2023). Mathematical thinking encompasses abilities such as analyzing problem structures, representing relationships symbolically or graphically, making and testing conjectures, selecting and adapting strategies, and constructing logical justifications (Genç et al., 2025). When faced with non-routine or contextualized problems, students with more advanced mathematical thinking are better able to identify the underlying mathematical ideas, to translate real-world situations into mathematical models, and to navigate among alternative solution paths (Baidoo and Ali, 2023). They can monitor the coherence of their reasoning, detect inconsistencies, and revise their approach when necessary, which increases the likelihood of arriving at valid and efficient solutions. By contrast, students with less developed mathematical thinking tend to rely on surface features, fixed templates, or trial-and-error procedures, which often fail when problems deviate from familiar formats (Elagha and Pellegrino, 2024). Thus, higher levels of mathematical thinking should naturally translate into stronger mathematical problem-solving performance (Torres-Peña et al., 2025). Hence, we posit:

*H3a*: Math self-efficacy positively predicts students’ mathematical problem-solving skills.

*H3b*: Mathematical thinking positively predicts students’ mathematical problem-solving skills.

Students with more adaptive personality profiles, such as higher conscientiousness and emotional stability tend to approach mathematical tasks in a more organized, persistent, and emotionally controlled manner (Dang et al., 2025), which can translate directly into better performance when solving complex problems under time pressure or examination conditions. Likewise, students who are more strongly motivated to learn mathematics are more inclined to devote effort, to engage seriously with practice tasks, and to seek out additional learning opportunities (e.g., extra exercises, online resources) (Asare et al., 2024), thereby accumulating more extensive experience with diverse problem types that supports higher problem-solving performance. In a similar vein, students who habitually regulate their learning by planning, monitoring, and adjusting strategies are better able to manage their time, select appropriate methods, and avoid common pitfalls during problem solving, which should benefit their actual test and classroom performance even when their self-beliefs and higher-order thinking are controlled (Polack and Miller, 2022). Taken together, these arguments suggest that personality traits, motivation to math, and self-regulated learning are not only antecedents of internal psychological states, but also proximal predictors of observable mathematical problem-solving skills (Říčan et al., 2022). Therefore, we propose the following hypotheses:

*H4a*: Personality traits positively predict students’ mathematical problem-solving skills.

*H4b*: Motivation to math positively predicts students’ mathematical problem-solving skills.

*H4c*: Self-regulated learning positively predicts students’ mathematical problem-solving skills.

Adaptive personality traits such as conscientiousness and emotional stability, higher levels of motivation to learn mathematics, and stronger self-regulated learning behaviors all increase the likelihood that students will experience success in mathematics, persist through difficulty, and interpret their progress in a constructive way (Sun and Liu, 2023). As suggested by social cognitive theory, these mastery experiences and positive interpretations are primary sources of self-efficacy beliefs (Jartitngarm, 2025). In other words, students who are more diligent, emotionally resilient, motivated, and self-regulated are more likely to build a robust sense of “I can do mathematics,” which then influences how they approach new problems (Moreira et al., 2020). Once established, higher math self-efficacy encourages students to engage more deeply with complex tasks, to persist when confronted with obstacles, and to employ more effective strategies during problem solving, thereby enhancing their actual performance (Voica et al., 2020; Zuo et al., 2024). Thus, it is reasonable to expect that personality traits, motivation to math, and self-regulated learning will not only have direct effects on mathematical problem-solving skills, but will also exert indirect effects through math self-efficacy as an intervening variable.

*H5a*: Math self-efficacy mediates the relationship between personality traits and mathematical problem-solving skills.

*H5b*: Math self-efficacy mediates the relationship between motivation to math and mathematical problem-solving skills.

*H5c*: Math self-efficacy mediates the relationship between self-regulated learning and mathematical problem-solving skills.

In addition to math self-efficacy, mathematical thinking is expected to function as a key cognitive mechanism linking personality traits, motivation to math, and self-regulated learning with students’ mathematical problem-solving skills (Landa et al., 2025). Adaptive personality profiles (e.g., higher conscientiousness and openness) and stronger motivation to learn mathematics increase the likelihood that students will engage more deeply with complex mathematical tasks, explore multiple solution strategies, and reflect on the underlying structures of problems (Armijo, 2025). Likewise, students who frequently regulate their learning by planning, monitoring, and adjusting their approaches are more likely to analyze relationships, generalize from examples, and justify their reasoning, all of which are core components of mathematical thinking (Ceballos et al., 2026). Over time, such dispositional and motivational advantages, together with self-regulated learning behaviors, are expected to foster more advanced mathematical thinking, which in turn enables students to better interpret problem situations, select appropriate strategies, and construct coherent solution paths. In this way, personality traits, motivation to math, and self-regulated learning may influence mathematical problem-solving not only directly, but also indirectly through their effects on mathematical thinking (Wang et al., 2025). Hence, we posit:

*H6a*: Mathematical thinking mediates the relationship between personality traits and mathematical problem-solving skills.

*H6b*: Mathematical thinking mediates the relationship between motivation to math and mathematical problem-solving skills.

*H6c*: Mathematical thinking mediates the relationship between self-regulated learning and mathematical problem-solving skills.

**Figure 1:**
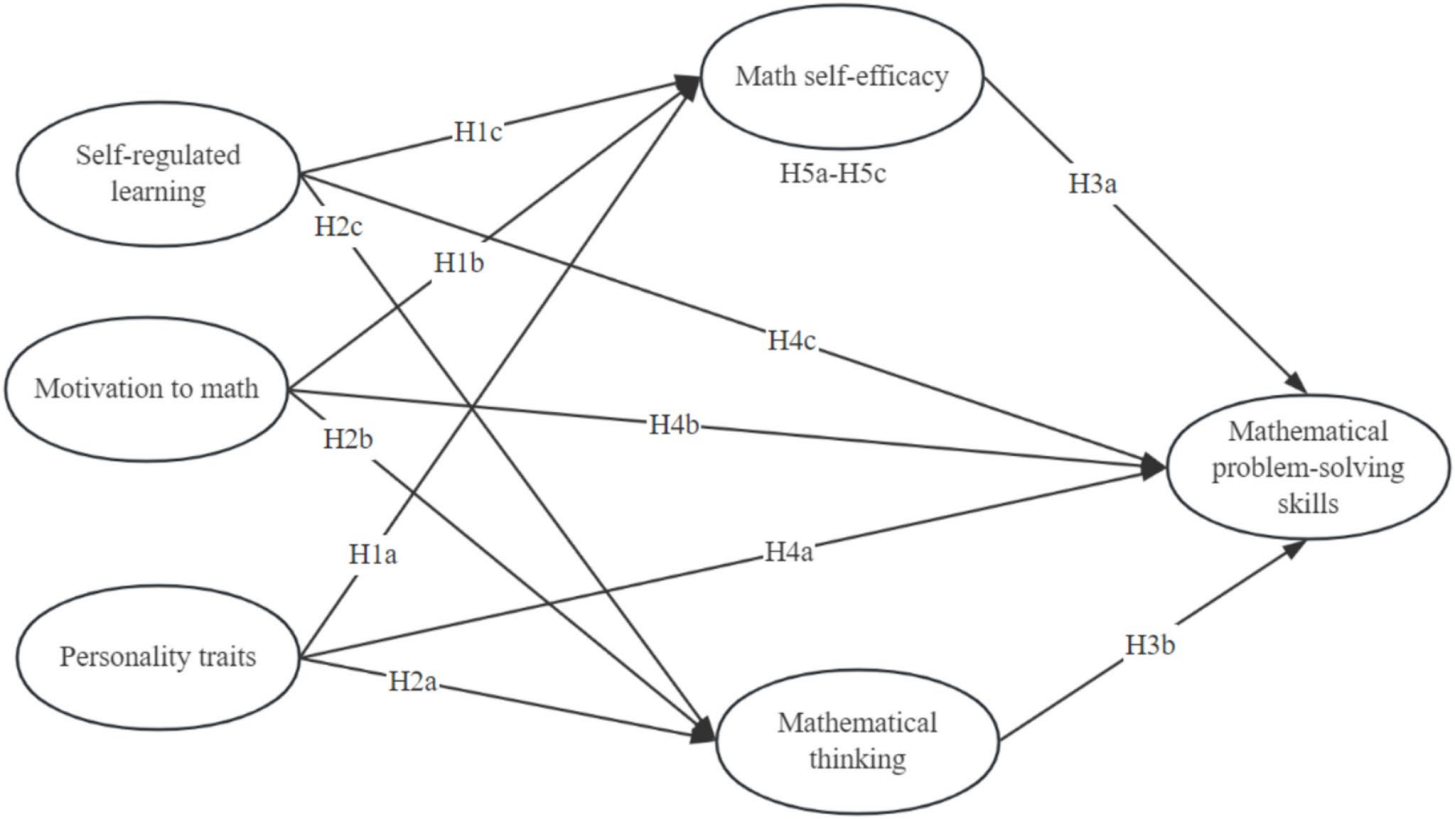
Conceptual framework.

Figure 1 presents the conceptual framework of this study. The model is designed not only to test a set of direct and indirect relationships, but also to evaluate the relative explanatory roles of distal personality traits and proximal learning processes in mathematical problem-solving. In this sense, the framework may also be read as a comparison between a trait-oriented account and a process-centered account of mathematical problem-solving, with the latter expected to show stronger explanatory relevance in the present high-performance context. In particular, it specifies two parallel mechanisms through which these antecedents may influence performance: an affective-belief pathway through math self-efficacy and a cognitive pathway through mathematical thinking. By distinguishing these two mechanisms and by treating higher-order mathematical problem-solving as the focal outcome, the framework extends prior research that has often emphasized either motivational beliefs, self-regulated learning processes, or broad individual differences in relative isolation. To address RQ1, we examine both the direct effects of personality traits, motivation to math, and self-regulated learning on students’ problem-solving skills (H4a–H4c), as well as their indirect effects via math self-efficacy and mathematical thinking (H5a–H5c, H6a–H6c). To address RQ2, we explicitly test whether math self-efficacy and mathematical thinking function as distinct mediating pathways in the proposed model.

Taken together, the reviewed literature suggests that the key issue is not whether personality traits, motivation, self-regulated learning, self-efficacy, and mathematical thinking are each individually relevant, because prior studies already indicate that they are. The more important unresolved issue is how these predictors should be theoretically ordered. The present study argues that broad personality traits are better understood as distal conditions, whereas motivation to math and self-regulated learning function as more proximal and modifiable learning processes. These processes are then expected to operate through two mathematics-specific mechanisms: an affective-belief pathway, reflected in mathematical self-efficacy, and a cognitive pathway, reflected in mathematical thinking. Such a framework allows the study to move beyond citation accumulation and toward a more critically integrated account of higher-order mathematical problem-solving, in which the central question is not simply which variables matter, but how their explanatory roles differ in level, mechanism, and contextual relevance.

## 3 Method

### 3.1 Data collection and sample

Data were collected in two rounds during the regular school semester in May, 2025 to July, 2025. In total, 1,400 questionnaires were returned from Grade 10 and Grade 11 students across the participating high schools in Shanghai. To ensure data quality, we conducted a series of screening procedures. First, cases with large portions of missing responses (e.g., more than 20% of items left unanswered) were removed. Second, questionnaires showing obvious patterned responding (e.g., the same option chosen for almost all items) or logically inconsistent answers were excluded. Third, extremely short completion times, which indicated that students were unlikely to have read the items carefully, were also used as a criterion for deletion. On the basis of these criteria, 217 questionnaires were excluded as low-quality responses. In addition, only students with complete information on all focal constructs and who could be matched across the two rounds of data collection were retained for the final analysis. This resulted in a final effective sample of 1,183 valid questionnaires.

### 3.2 Measurement

All focal constructs were measured with multi-item instruments adapted from recent, well-validated scales in mathematics education and self-regulation research. Personality traits were assessed with a shortened Big Five inventory for adolescents (12 items), adapted from the Big Five Inventory–2 and its short forms (Soto and John, 2017), focusing on conscientiousness, emotional stability, and openness to experience as key dispositions relevant for learning; students rated how well each statement described them on a five-point Likert scale (1 = strongly disagree to 5 = strongly agree). Motivation to math was measured by a 10-item scale adapted from recent expectancy–value–based mathematics motivation questionnaires for upper-secondary students (Kirkham et al., 2023; Mayerhofer et al., 2024; Treacy et al., 2024), capturing students’ intrinsic interest in mathematics, perceived importance and usefulness of math, and mastery-oriented learning goals. Self-regulated learning in mathematics was assessed with an 11-item scale adapted from contemporary self-regulated learning instruments developed for secondary and postsecondary learners (Liu et al., 2025; Shi and Yang, 2025), targeting three core dimensions: planning (e.g., setting goals for math study), monitoring (e.g., checking understanding during problem solving), and strategy regulation (e.g., adjusting methods and effort when encountering difficulties). Math self-efficacy was measured using eight items adapted from mathematics self-efficacy scales commonly used with high school students (Shimizu, 2025; Yu et al., 2023), reflecting students’ confidence in understanding difficult math content, solving challenging problems, and performing well on math tests.

Mathematical thinking and mathematical problem-solving skills were measured with curriculum-based performance tasks aligned with recent international assessment frameworks for mathematical literacy and reasoning (Wang, 2025; Xu et al., 2025): an eight-item non-routine/application test assessing higher-order mathematical thinking (reasoning, modeling, and justification) and a six-item contextual word-problem test assessing problem-solving performance in exam-like situations; both tests were scored with analytic rubrics and total scores were used as indicators of the respective constructs. To ensure an operational distinction between the two performance-based constructs during the task design phase, the mathematical thinking tasks and the mathematical problem-solving tasks were developed with different measurement emphases rather than being separated merely by difficulty level. More specifically, the mathematical thinking tasks were designed to elicit the quality of students’ reasoning, abstraction/modeling, generalization, and justification, even when a response did not take the form of a fully completed contextual solution. By contrast, the mathematical problem-solving tasks were designed to assess students’ ability to interpret contextualized mathematical situations, select and execute an appropriate strategy, and arrive at an accurate and explainable solution under exam-like conditions. In this sense, mathematical thinking was operationalized primarily as the quality of higher-order mathematical cognition, whereas mathematical problem-solving was operationalized primarily as successful performance in contextualized task completion. Although the two constructs were expected to be related because stronger mathematical thinking can support more effective problem-solving, they were differentiated by intended task focus and scoring emphasis from the outset of instrument design.

In the present study, these performance-based tasks were modeled as reflective indicators because each item was designed to capture observable manifestations of an underlying latent capability rather than to represent independent components that jointly define the construct. More specifically, the mathematical thinking items were intended to reflect a common higher-order capacity involving reasoning, modeling, and justification, whereas the mathematical problem-solving items were designed to reflect a broader latent ability to solve contextualized mathematical problems. Accordingly, although performance-based constructs may in some studies be specified formatively or treated as composites, the current study treated item performance as arising from students’ underlying competence levels and therefore adopted a reflective measurement logic. In addition, several background variables were recorded as observed indicators (gender, grade level, academic track, parental education, cram school participation, only-child status, school area), and prior math achievement was measured by students’ self-reported score (out of 150 points) in their most recent mathematics examination and later included as a control variable in the structural model.

The full wording of all self-report items is provided in Appendix Table A2, and a summary of the analytic rubric criteria and scoring procedures for the performance-based tasks is provided in Appendix Table A3 to improve measurement transparency and support replication.

### 3.3 Data analysis

Descriptive statistics, preliminary screening, and data preparation were conducted in IBM SPSS Statistics 27, and the PLS-SEM analyses were performed in SmartPLS 4.0. Means, standard deviations, skewness, and kurtosis were computed for all major continuous variables to assess distributional properties. The hypotheses were tested using partial least squares structural equation modeling (PLS-SEM), as the study focused on comparing the explanatory relevance of competing predictors and mediating mechanisms across multiple endogenous constructs rather than on covariance-model fit alone (Huang, 2021). For the measurement model, indicator reliability was assessed through outer loadings, internal consistency through Cronbach’s alpha, rho_A, and composite reliability, convergent validity through average variance extracted (AVE), and discriminant validity through the Fornell–Larcker criterion, HTMT, and cross-loadings (Adamson and Prion, 2013; Fornell and Larcker, 1981). Because several constructs were measured by self-report, common method bias was also examined; Harman’s single-factor test showed that the first unrotated factor accounted for 28.64% of the total variance, suggesting that common method bias was unlikely to be a serious concern.

For the structural model, path coefficients were estimated using bootstrapping with 5,000 resamples and two-tailed tests at the 0.05 significance level (Kock, 2015). The mediating roles of math self-efficacy and mathematical thinking were tested by examining indirect effects and their bootstrapped confidence intervals, with both constructs specified as parallel mediators. Model performance was evaluated using R^2^, f^2^, and Q^2^, and out-of-sample predictive power was further assessed through PLSpredict. In addition, SRMR was reported as a supplementary model-fit index, and variance inflation factor (VIF) values were inspected to assess multicollinearity. All control variables were entered as observed covariates predicting math self-efficacy, mathematical thinking, and mathematical problem-solving skills. A reduced structural model excluding control variables and auxiliary specifications was also estimated as a robustness check.

### 3.4 Pilot study

Prior to the main survey, a pilot study was conducted to examine the clarity and reliability of the instruments. A sample of 50 Grade 10 students from a Shanghai public high school was randomly selected and completed the full questionnaire and a shortened version of the mathematical performance tasks. After the administration, students were invited to provide feedback on item wording, length, and overall comprehensibility. Internal consistency estimates for the multi-item scales were satisfactory, with Cronbach’s alpha values ranging from 0.82 to 0.91 across personality traits, motivation to math, self-regulated learning, and math self-efficacy. A few items with ambiguous wording or overlapping meanings were slightly revised (e.g., simplifying expressions and adding concrete examples in the self-regulated learning and motivation items), but no major changes were made to the underlying constructs or the structure of the tests. The revised instruments were then used in the large-scale data collection with 1,183 students.

## 4 Results

### 4.1 Descriptive statistical analysis

A total of 1,183 valid questionnaires were retained for analysis. As shown in Table 1, the sample demonstrates a generally balanced demographic structure. In terms of gender, 578 students (48.9%) were female and 605 (51.1%) were male. Regarding grade level, 621 students (52.5%) were in Grade 10 and 562 (47.5%) were in Grade 11, indicating a relatively even distribution across the two grade cohorts.

**Table 1:** Participant information.

Variable	Category	*n*	%/Note
Gender	Female	578	48.9
	Male	605	51.1
Grade level	Grade 10	621	52.5
	Grade 11	562	47.5
Academic track	Liberal arts	468	39.6
	Science	715	60.4
Parental education	Below bachelor’s degree	117	9.9
	Bachelor’s degree	457	38.6
	Master’s degree	430	36.3
	Doctoral degree	179	15.1
Cram school participation	No	426	36.0
	Yes	757	64.0
Only-child status	No	547	46.2
	Yes	636	53.8
School area	Urban schools	746	63.1
	Suburban schools	437	36.9
Age	Mean (SD)	16.53	0.67
	Range	15–18	—
Prior math achievement	Mean (SD)	103.00	—
	Range	82–147	—

Participants were aged 15–18 years, with a mean age of 16.53 years (SD = 0.67), which is consistent with the typical age range of upper secondary students. In terms of academic track, 468 students (39.6%) were in the liberal arts stream and 715 (60.4%) were in the science stream, suggesting a higher representation of science-track students in the participating schools.

Family socioeconomic background was proxied by parental educational attainment. Specifically, 117 students (9.9%) reported that their parents’ highest education level was below a bachelor’s degree, whereas 457 (38.6%) reported a bachelor’s degree, 430 (36.3%) reported a master’s degree, and 179 (15.1%) reported a doctoral degree. Overall, the parental education level in this sample is relatively high, with approximately 90% of students coming from families in which parental education reaches at least the undergraduate level.

With respect to learning support experiences, 757 students (64.0%) reported participating in extracurricular mathematics tutoring, while 426 (36.0%) did not. In addition, 636 students (53.8%) were only children, and 547 (46.2%) came from non–only-child families. In terms of school location, 746 students (63.1%) attended urban regular high schools, and 437 (36.9%) attended suburban regular high schools, which helps capture potential contextual differences associated with school area.

Prior mathematics achievement was measured using students’ self-reported scores from their most recent mathematics examination. The mean score was 103.00 (SD = 16.00), ranging from 82 to 147 (out of 150), indicating substantial variation in mathematics performance within the sample. These background variables (gender, grade level, academic track, parental education, cram school participation, only-child status, school area, age, and prior math achievement) will be included as control variables in subsequent structural equation modeling analyses.

**Figure 2:**
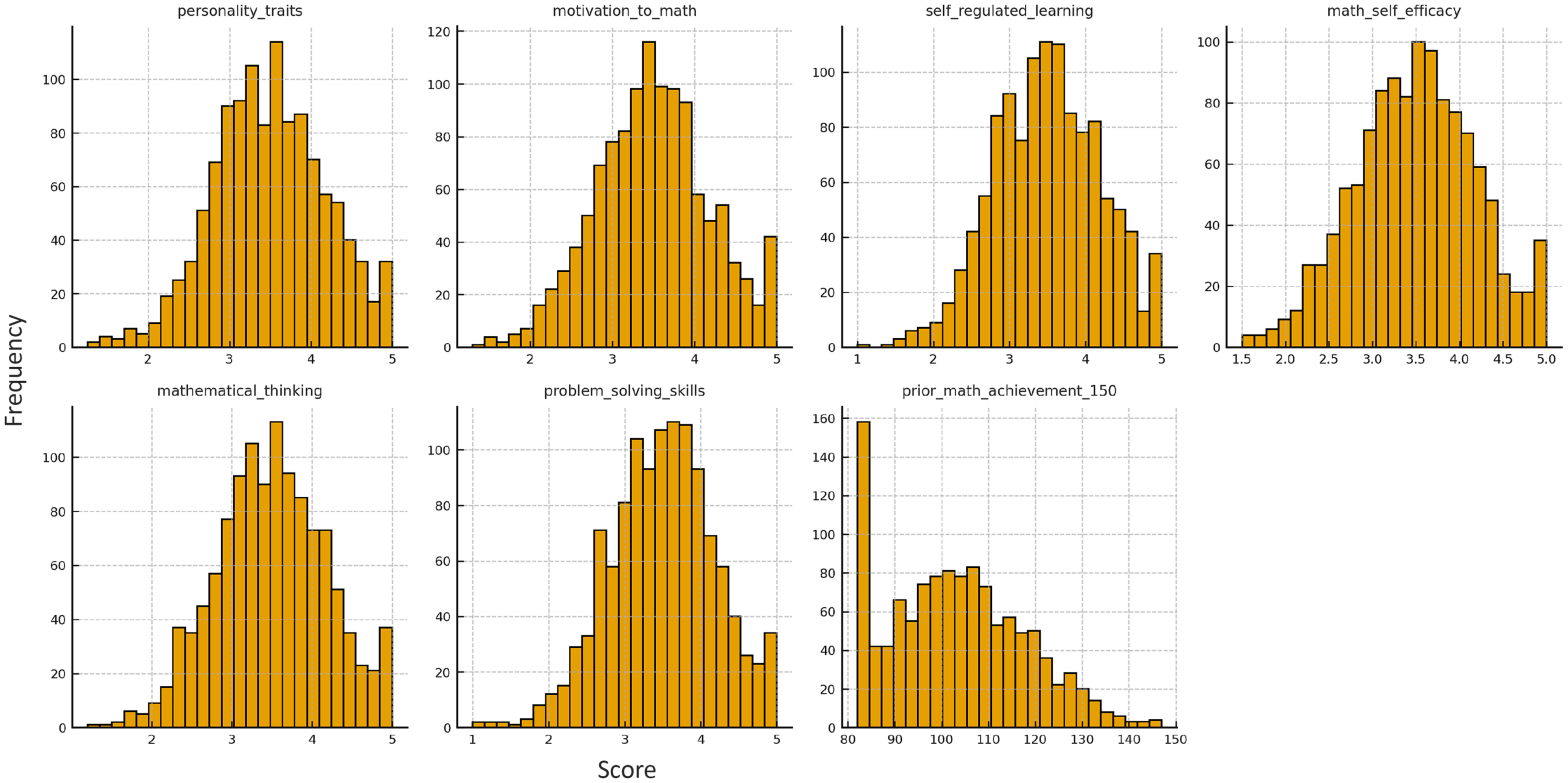
Distributions of main study variables.

Figure 2 presents the frequency distributions of key continuous variables in this study, including personality traits, mathematical learning motivation, self-regulated learning, mathematical self-efficacy, mathematical thinking, mathematical problem-solving ability, and prior mathematics scores. The data reveals that all six psychological variables (excluding mathematics scores) cluster predominantly in the upper-middle range of the 1-5-point scale, exhibiting a unimodal distribution pattern with near-normal characteristics. The data show only minor skewness and kurtosis, with no significant outliers or extreme deviations. The prior mathematics scores (out of 150 points) also follow a bell-shaped distribution, primarily concentrated between 90–120 points, with relatively fewer high and low-score samples at both ends. Collectively, these variables demonstrate strong continuity and near-normal characteristics, providing a solid distributional foundation for subsequent correlation analyses and structural equation modeling.

### 4.2 Measurement model

The psychometric properties of the reflective constructs were examined before testing the structural relationships. As shown in Table 2, all retained indicators loaded strongly on their intended constructs. Standardized outer loadings ranged from approximately 0.70 to 0.88 across all items, thus exceeding the recommended threshold of 0.70 and indicating satisfactory indicator reliability. The internal consistency of the six latent variables was high: composite reliability (CR) values ranged from 0.915 to 0.954 for personality traits, motivation to math, self-regulated learning, math self-efficacy, mathematical thinking, and mathematical problem-solving skills. In addition, the average variance extracted (AVE) for all constructs was above 0.50 (AVE = 0.629–0.652), providing evidence of adequate convergent validity.

**Table 2:** Factor loadings, composite reliability, and AVE for the measurement model.

Construct	Item	Loading	Composite_Reliability	AVE
PT	PT1	0.71	0.953	0.629
	PT2	0.71		
	PT3	0.74		
	PT4	0.75		
	PT5	0.76		
	PT6	0.80		
	PT7	0.81		
	PT8	0.81		
	PT9	0.83		
	PT10	0.84		
	PT11	0.86		
	PT12	0.88		
MOT	MOT1	0.71	0.947	0.64
	MOT2	0.75		
	MOT3	0.78		
	MOT4	0.77		
	MOT5	0.78		
	MOT6	0.80		
	MOT7	0.81		
	MOT8	0.85		
	MOT9	0.85		
	MOT10	0.88		
SRL	SRL1	0.75	0.954	0.652
	SRL2	0.74		
	SRL3	0.76		
	SRL4	0.78		
	SRL5	0.78		
	SRL6	0.82		
	SRL7	0.81		
	SRL8	0.84		
	SRL9	0.85		
	SRL10	0.86		
	SRL11	0.88		
MSE	MSE1	0.70	0.933	0.636
	MSE2	0.73		
	MSE3	0.76		
	MSE4	0.80		
	MSE5	0.79		
	MSE6	0.84		
	MSE7	0.86		
	MSE8	0.88		
MT	MT1	0.73	0.937	0.651
	MT2	0.76		
	MT3	0.77		
	MT4	0.79		
	MT5	0.82		
	MT6	0.83		
	MT7	0.86		
	MT8	0.88		
PS	PS1	0.75	0.915	0.644
	PS2	0.75		
	PS3	0.77		
	PS4	0.81		
	PS5	0.84		
	PS6	0.88		

Discriminant validity was assessed using the Fornell–Larcker criterion, HTMT ratios, and indicator cross-loadings. As reported in Table 3, the square roots of the AVE values (diagonal elements; 0.79–0.81) were greater than the corresponding correlations between each construct and all other constructs (off-diagonal elements; |*r*| = 0.26–0.58). In addition, all HTMT values were below the recommended threshold of 0.85, further supporting discriminant validity (Table 4). Cross-loading inspection also showed that each indicator loaded highest on its intended construct. For reasons of space, the complete cross-loading matrix is provided in Appendix Table A1. Taken together, these results indicate that the measurement model demonstrates satisfactory indicator reliability, internal consistency, convergent validity, and discriminant validity, and is therefore suitable for subsequent evaluation of the structural model. The moderate correlation between mathematical thinking and mathematical problem-solving (*r* = 0.58) was theoretically expected, because students with stronger reasoning, modeling, and justification abilities are also more likely to perform well on contextualized mathematical problems. At the same time, the two constructs were not treated as interchangeable. As described in the measurement design, mathematical thinking tasks were intended to capture the quality of higher-order mathematical cognition, whereas mathematical problem-solving tasks were intended to capture students’ successful interpretation and solution of contextualized problem situations. Their empirical association therefore reflects theoretical relatedness rather than construct redundancy, which is also consistent with the acceptable discriminant-validity evidence shown in the Fornell–Larcker and HTMT results. It should also be noted that the core model does not rely exclusively on self-reported indicators, because mathematical thinking and mathematical problem-solving were measured through performance-based tasks. This mixed-source measurement design helps reduce, though not fully eliminate, concerns regarding common method variance.

**Table 3:** Fornell–Larcker discriminant validity of the latent constructs.

Construct	PT	MOT	SRL	MSE	MT	PS
PT	**0.79**					
MOT	0.30	**0.80**				
SRL	0.35	0.50	**0.81**			
MSE	0.30	0.48	0.46	**0.80**		
MT	0.28	0.42	0.41	0.53	**0.81**	
PS	0.26	0.39	0.35	0.40	0.58	0.80

Bold values on the diagonal are the square roots of the average variance extracted (AVE). Discriminant validity is supported when the square root of AVE for each construct is greater than its correlations with other constructs.

**Table 4:** HTMT ratios of the latent constructs.

Construct	PT	MOT	SRL	MSE	MT	PS
PT	—	0.354	0.401	0.347	0.322	0.298
MOT	0.354	—	0.561	0.536	0.478	0.441
SRL	0.401	0.561	—	0.519	0.468	0.413
MSE	0.347	0.536	0.519	—	0.601	0.458
MT	0.322	0.478	0.468	0.601	—	0.648
PS	0.298	0.441	0.413	0.458	0.648	—

All HTMT values were below the recommended threshold of 0.85, supporting discriminant validity.

### 4.3 Structural model and hypothesis testing

As shown in Table 5, the structural model demonstrated substantial explanatory power. Using personality traits, motivation to math, and self-regulated learning as antecedent variables, the model explained 54% of the variance in mathematical self-efficacy (R^2^ = 0.54), 57% of the variance in mathematical thinking (R^2^ = 0.57), and 63% of the variance in mathematical problem-solving ability (R^2^ = 0.63). According to commonly used guidelines, these values indicate a moderately high level of explanatory power. In addition, the model showed acceptable approximate fit in PLS-SEM terms, with an SRMR value of 0.061, which was below the recommended cutoff of 0.08.

**Table 5:** Direct effects of the structural model and hypothesis testing.

Path	Hypothesis	Beta	T	*p*	f2	Result
PT → MSE	H1a	0.1	1.86	0.063	0.012	Rejected
MOT → MSE	H1b	0.38	7.42	0	0.185	Supported
SRL → MSE	H1c	0.33	6.51	0	0.146	Supported
PT → MT	H2a	0.07	1.42	0.156	0.008	Rejected
MOT → MT	H2b	0.32	6.18	0	0.139	Supported
SRL → MT	H2c	0.41	7.95	0	0.215	Supported
MSE → PS	H3a	0.29	5.87	0	0.112	Supported
MT → PS	H3b	0.36	7.24	0	0.168	Supported
PT → PS	H4a	0.05	1.31	0.191	0.006	Rejected
MOT → PS	H4b	0.14	2.72	0.007	0.027	Supported
SRL → PS	H4c	0.18	3.34	0.001	0.039	Supported

Supplementary structural diagnostics are reported in Tables 6–8. As summarized in Table 6, all VIF values remained within an acceptable range, suggesting that multicollinearity was not a serious concern, and the Q^2^ values indicated satisfactory predictive relevance for the endogenous constructs. As detailed in Table 7, the PLSpredict results showed that the PLS model yielded lower prediction errors than the linear-model benchmark for most indicators across mathematical self-efficacy, mathematical thinking, and mathematical problem-solving, supporting moderate predictive power at the construct level rather than merely acceptable in-sample explanation. Table 8 reports the robustness analysis based on a reduced structural model in which background control variables and non-essential auxiliary specifications were removed while the core theoretical paths were retained. The substantive pattern of the main theoretical paths remained unchanged, suggesting that the principal conclusions were stable rather than dependent on a single specification.

**Table 6:** Supplementary structural model diagnostics.

Diagnostic	Value/Range	Interpretation
SRMR	0.061	Acceptable approximate fit
VIF	1.21–2.46	No serious multicollinearity
Q^2^ (MSE)	0.331	Predictive relevance supported
Q^2^ (MT)	0.362	Predictive relevance supported
Q^2^ (PS)	0.401	Predictive relevance supported
PLSpredict summary	PLS errors lower than LM benchmark for most indicators	Adequate out-of-sample predictive performance
Robustness check	Substantive pattern unchanged	Results stable

**Table 7:** Summary of PLSpredict results for the endogenous constructs.

Construct	Indicator set	PLS lower RMSE/MAE than LM benchmark	Predictive assessment
MSE	MSE1–MSE8	7 of 8 indicators	Moderate predictive power
MT	MT1–MT8	6 of 8 indicators	Moderate predictive power
PS	PS1–PS6	5 of 6 indicators	Moderate predictive power

**Table 8:** Robustness check based on a reduced structural model.

Path pattern	Main model	Reduced model	Stability
MOT → MSE	Significant	Significant	Stable
SRL → MSE	Significant	Significant	Stable
MOT → MT	Significant	Significant	Stable
SRL → MT	Significant	Significant	Stable
MSE → PS	Significant	Significant	Stable
MT → PS	Significant	Significant	Stable
PT → PS	Non-significant	Non-significant	Stable

In the path toward mathematical self-efficacy, both motivation to math (*β* = 0.38, *t* = 7.42, *p* < 0.001, f^2^ = 0.185) and self-regulated learning (*β* = 0.33, *t* = 6.51, *p* < 0.001, f^2^ = 0.146) showed significant positive predictive effects, supporting H1b and H1c. In contrast, the direct effect of personality traits on mathematical self-efficacy was not significant (*β* = 0.10, *t* = 1.86, *p* = 0.063, f^2^ = 0.012), and H1a was not supported. This indicates that, after controlling for the other variables, students’ mathematical self-efficacy was predicted mainly by their motivation to math and self-regulated learning, whereas the direct contribution of personality traits was comparatively weak.

Regarding the ability to solve mathematical problems, both mathematical self-efficacy (*β* = 0.29, *t* = 5.87, *p* < 0.001, f^2^ = 0.112) and mathematical thinking (*β* = 0.36, *t* = 7.24, *p* < 0.001, f^2^ = 0.168) have significant positive effects. Hypotheses H3a and H3b are supported. At the same time, motivation to math (*β* = 0.14, *t* = 2.72, *p* = 0.007, f^2^ = 0.027) and self-regulated learning (*β* = 0.18, *t* = 3.34, *p* = 0.001, f^2^ = 0.039) retained statistically significant direct effects on problem-solving performance, although the corresponding effect sizes were small. This suggests that their substantive importance lies less in strong standalone direct impacts and more in their broader role as upstream learning-process variables that influence problem-solving both directly and indirectly through mathematical self-efficacy and mathematical thinking; The direct effect of personality traits on problem-solving ability is not significant (*β* = 0.05, *t* = 1.31, *p* = 0.191, f^2^ = 0.006), and H4a is not supported. Overall, the results indicate that motivation to math and self-regulated learning indirectly promote problem-solving by enhancing mathematical self-efficacy and mathematical thinking, while retaining a certain direct effect; Personality traits exert more influence through indirect pathways, and their direct effects are relatively limited.

Table 6 summarizes the supplementary structural diagnostics for the present model, including approximate model fit (SRMR), multicollinearity diagnostics (VIF), predictive relevance (Q^2^), an overall PLSpredict summary, and the robustness-check result. Together, these statistics indicate that the model was not only explanatory in-sample but also acceptable in terms of collinearity control, predictive relevance, and specification stability.

As detailed in Table 7, the PLSpredict results showed that the PLS model yielded lower prediction errors than the linear benchmark for most indicators across mathematical self-efficacy, mathematical thinking, and mathematical problem-solving, supporting moderate predictive power at the construct level rather than merely acceptable in-sample explanation.

Table 8 reports the robustness analysis based on the reduced structural model. The purpose of this table is not to re-estimate every parameter in full detail, but to show that the substantive pattern of the core theoretical paths remained unchanged after simplifying the model specification. This supports the stability of the main conclusions.

**Figure 3:**
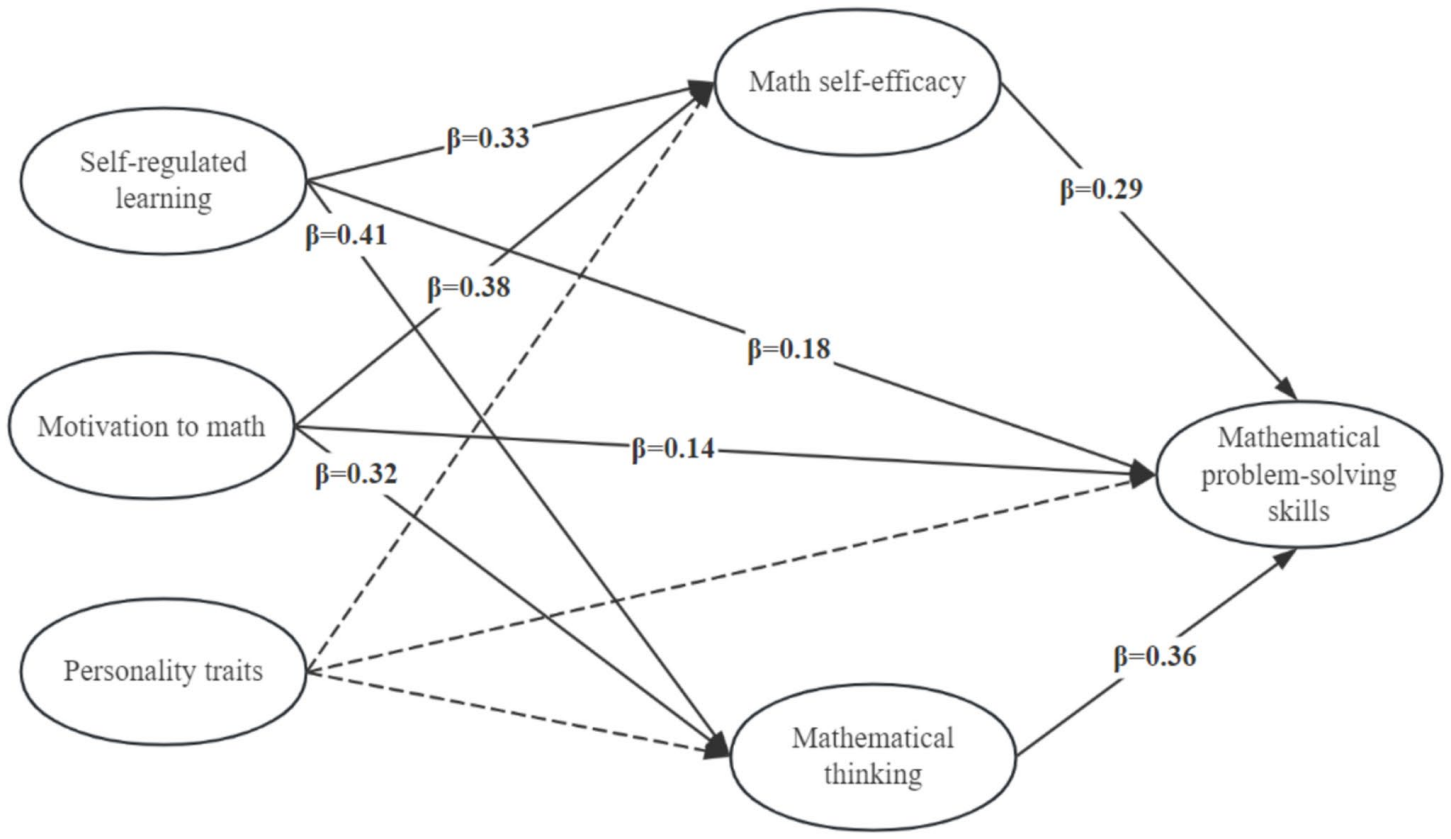
Motivation–self-regulation model of mathematical problem-solving through self-efficacy and mathematical thinking.

Figure 3 presents a model that explains the skill of solving mathematical problems.

To test the mediating effect of mathematical self-efficacy and mathematical thinking on the relationship between various antecedent variables and mathematical problem-solving ability, this study used bootstrap (5,000 resampling) in PLS-SEM to estimate indirect effects and their 95% confidence intervals. The results are shown in Table 9.

**Table 9:** Indirect effects of the structural model via math self-efficacy and mathematical thinking.

Indirect_path	Mediator	Hypothesis	Indirect_beta	T	*p*	LL_95CI	UL_95CI	Result
PT → MSE → PS	MSE	H5a	0.03	1.58	0.114	−0.01	0.08	Rejected
MOT → MSE → PS		H5b	0.11	4.82	0	0.06	0.17	Supported
SRL → MSE → PS		H5c	0.1	4.39	0	0.05	0.16	Supported
PT → MT → PS	MT	H6a	0.03	1.49	0.137	−0.01	0.07	Rejected
MOT → MT → PS		H6b	0.12	5.03	0	0.07	0.18	Supported
SRL → MT → PS		H6c	0.15	5.76	0	0.1	0.22	Supported

Firstly, from the indirect pathway of mathematical self-efficacy, both motivation to math (MOT) and self-regulated learning (SRL) exhibit significant positive indirect effects. Specifically, the indirect effect coefficient of MOT → MSE → PS is *β* = 0.11, *t* = 4.82, *p* < 0.001, with a 95% confidence interval of [0.06, 0.17], excluding 0, assuming that H5b is supported; The indirect effect of SRL → MSE → PS is *β* = 0.10, *t* = 4.39, *p* < 0.001, with a 95% confidence interval of [0.05, 0.16], also excluding 0, supporting H5c. In contrast, the indirect effect of personality traits (PT) on mathematical self-efficacy was not significant (*β* = 0.03, *t* = 1.58, *p* = 0.114, 95% CI [−0.01, 0.08]), and H5a was rejected. This indicates that, under the control of other variables, motivation to math and self-regulated learning can enhance students’ mathematical self-efficacy, further promoting their problem-solving performance, while the influence of personality traits on problem-solving is not significantly transmitted through this emotional pathway.

Secondly, from the perspective of indirect pathways through mathematical thinking, both motivation to math and self-regulated learning exhibit significant mediating effects. The indirect effect of MOT → MT → PS is *β* = 0.12, *t* = 5.03, *p* < 0.001, 95% CI [0.07, 0.18], supporting H6b; The indirect effect of SRL → MT → PS is *β* = 0.15, *t* = 5.76, *p* < 0.001, 95% CI [0.10, 0.22], supporting H6c. In contrast, the indirect effect of PT → MT → PS is still not significant (*β* = 0.03, *t* = 1.49, *p* = 0.137, 95% CI [−0.01, 0.07]), assuming H6a is rejected. This indicates that motivation to math and self-regulated learning not only enhance students’ mathematical self-efficacy, but also indirectly improve their problem-solving skills by strengthening their mathematical thinking abilities. However, the influence of personality traits on problem-solving has not been significantly affected through the cognitive pathway of mathematical thinking.

By combining direct and indirect effects, it can be seen that for motivation to math and self-regulated learning, the direct path towards problem-solving ability remains significant even after incorporating mediating variables (see Table 9). At the same time, the indirect effects through mathematical self-efficacy and mathematical thinking are also significant, thus presenting a pattern of partial mediation: on the one hand, motivation and self-regulated learning can directly predict students’ mathematical problem-solving performance; On the other hand, they also indirectly play a role through two mediating pathways: enhancing self-efficacy and mathematical thinking. In contrast, the direct effect and two indirect effects of personality traits on problem-solving ability are not significant, indicating that their effects are mainly “absorbed” by more proximal learning variables (motivation, self-regulation, self-efficacy, and mathematical thinking), and a clear mediating chain has not been formed in this model.

Overall, the results in Table 9 support the “motivation self-regulation cognitive/affective mechanism” pathway proposed in this study: motivation to math and self-regulated learning significantly enhance students’ mathematical self-efficacy (affective mechanism) and mathematical thinking (cognitive mechanism) by simultaneously improving their mathematical problem-solving ability, while the mediating role of personality traits in this pathway is relatively limited.

To further clarify the mediation structure, the total effects were decomposed into direct and total indirect components. The decomposition showed that the overall effect of self-regulated learning on mathematical problem-solving was the strongest among the antecedent variables, followed by motivation to math, whereas the total effect of personality traits remained comparatively weak. For motivation to math and self-regulated learning, a substantial proportion of the total effects was transmitted through indirect pathways, indicating that their contribution to problem-solving depended not only on direct prediction but also on the combined operation of the two parallel mediators. This pattern further supports the interpretation that mathematical self-efficacy and mathematical thinking function as meaningful transmission mechanisms linking upstream learning processes to mathematical problem-solving performance (Table 10).

**Table 10:** Decomposition of direct, indirect, and total effects on mathematical problem-solving.

Predictor	Direct effect on PS	Total indirect effect	Total effect
PT	0.05	0.06	0.11
MOT	0.14	0.23	0.37
SRL	0.18	0.25	0.43

From the perspective of substantive contribution within the structural model, mathematical thinking emerged as the strongest direct predictor of mathematical problem-solving, with a moderate effect size (f^2^ = 0.168), while mathematical self-efficacy also showed a meaningful direct contribution (f^2^ = 0.112). By contrast, the direct effects of motivation to math and self-regulated learning on problem-solving were statistically significant but small in magnitude (f^2^ = 0.027 and 0.039, respectively). This suggests that their importance lies less in strong standalone direct effects and more in their role as upstream learning-process variables that shape performance both directly and indirectly through mathematical self-efficacy and mathematical thinking. Taken together, the pattern of direct and indirect effects suggests that self-regulated learning may play the strongest upstream role in the present model, followed by motivation to math. In contrast, the direct and mediated effects of personality traits were not significant, indicating that, in the present context, the contribution of broad personality differences to mathematical problem-solving was comparatively limited once more proximal learning-process variables were taken into account.

A comparison of the indirect effects further showed that, for both motivation to math and self-regulated learning, the pathway through mathematical thinking was slightly stronger than the pathway through mathematical self-efficacy. This suggests that, in the present context, the cognitive transmission route may be somewhat more prominent than the affective-belief route, although both mechanisms were meaningful.

## 5 Discussion

This study examined how personality traits, motivation to math, and self-regulated learning were associated with high school students’ mathematical problem-solving in Shanghai, while also clarifying the mediating roles of mathematical self-efficacy and mathematical thinking. The main theoretical contribution lies not simply in showing that motivation and self-regulated learning were significant, but in clarifying the relative explanatory ordering of broad traits and more proximal learning processes within a mathematics-specific framework. After prior mathematics achievement and background variables were controlled, the explanatory center of the model shifted toward mathematics-specific and modifiable learning processes rather than broad personality traits. In this sense, the findings support a process-centered rather than trait-centered account of higher-order mathematical problem-solving.

The results support a dual-mechanism interpretation in which mathematical self-efficacy and mathematical thinking functioned as two distinct pathways linking upstream learning processes to problem-solving performance. This pattern is theoretically meaningful because it suggests that mathematical problem-solving should not be reduced either to confidence alone or to cognition in isolation. Rather, successful performance appears to depend on the joint operation of students’ belief that they can engage effectively with mathematical tasks and their capacity to reason, model, and justify solutions. In this respect, the findings extend prior discussions that have often emphasized either self-efficacy-related beliefs or higher-order cognitive engagement more separately (Genç et al., 2025; Zuo et al., 2024).

Among the upstream predictors, self-regulated learning occupied a particularly important position. It was associated not only with mathematical problem-solving directly, but also with both mathematical self-efficacy and mathematical thinking. This pattern is consistent with evidence that effective self-regulation supports performance in demanding academic contexts, including mathematics (Lee et al., 2025), and with recent reviews highlighting its close links to metacognitive monitoring, strategic persistence, and adaptive adjustment (Waheed et al., 2025). In mathematics education specifically, self-regulatory practices have increasingly been viewed as central to engagement with non-routine tasks rather than as merely supplementary study habits (Hadwin et al., 2025). What the present study adds is a clearer positioning of self-regulated learning as a proximal learning-process variable that links confidence, thinking quality, and performance within one explanatory sequence.

Motivation was positively associated with mathematical self-efficacy, mathematical thinking, and mathematical problem-solving, suggesting that students’ willingness to engage meaningfully with mathematics remains important even after prior achievement and background factors are considered. This is consistent with expectancy-value research showing that students with stronger interest, value beliefs, and mastery-oriented engagement tend to participate more actively and perform better in mathematics-related tasks (Jiang and Zhang, 2023), as well as with recent findings linking mathematics motivation to participation profiles and achievement trajectories in secondary education (Gülşen Turgut and Bakır, 2025). In this study, motivation retained both direct and indirect relevance, implying that problem-solving differences depend not only on procedural knowledge, but also on students’ willingness to invest effort, persist through difficulty, and treat mathematics as meaningful.

The two mediators, however, did not contribute equally. Although mathematical self-efficacy was a meaningful direct predictor and mediator, the indirect pathway through mathematical thinking was slightly stronger for both motivation to math and self-regulated learning. This result suggests that, in the present context, the translation of motivational and regulatory strengths into successful problem-solving depends especially on the quality of students’ reasoning, abstraction, and justification. This is consistent with work emphasizing that such higher-order thinking is central to performance on complex and non-routine mathematical tasks (Ceballos et al., 2026) and to deep mathematical understanding more broadly (Chen, 2024). At the same time, the role of mathematical self-efficacy remains important, in line with findings that students with stronger mathematics self-efficacy tend to persist more and engage more effectively with difficult tasks (Zakariya, 2022), particularly under conditions of uncertainty and temporary failure (Chirove, 2023). Taken together, these results suggest that affective-belief and cognitive pathways are both consequential, but that the cognitive pathway may be more proximal to higher-order problem-solving performance.

The most theoretically consequential finding, however, concerns the comparatively weak role of personality traits once more proximal mathematics-specific variables were included in the model. Personality traits did not significantly predict mathematical self-efficacy, mathematical thinking, or mathematical problem-solving, nor did they show significant indirect effects through the two mediators. This result stands in partial tension with broader evidence showing that personality traits, especially conscientiousness, often have small to moderate associations with academic performance (Mammadov, 2022), and with work suggesting that the Big Five may shape achievement indirectly through self-regulation, engagement, or emotional adjustment (Fuente et al., 2024). However, the present findings should not be read as evidence that personality is irrelevant to mathematics learning in general. Rather, they suggest that the explanatory role of personality may become attenuated when mathematics-specific motivational, regulatory, affective, and cognitive processes are represented directly in the same model. This interpretation is compatible with work showing that trait-based influences on achievement are often modest and context-dependent even when statistically detectable (Ackerman and Kanfer, 2025).

This attenuation may be especially meaningful in the Shanghai context. In a high-performance educational environment characterized by strong instructional expectations, examination pressure, and routinized academic conduct, the visible expression of broad dispositional differences may be compressed. Under such conditions, students may appear similarly diligent or compliant in outward behavior, while the more decisive differences lie in how effectively they sustain motivation, regulate learning, evaluate their own capability, and engage in higher-order mathematical thinking. From this perspective, the findings suggest that the apparent strength of personality-based explanations is context-sensitive rather than universally stable. In highly structured and performance-oriented settings, proximal learning processes may be more informative than broad traits for explaining domain-specific outcomes such as higher-order mathematical problem-solving.

Overall, the findings support a layered, process-centered account of mathematical problem-solving. In the present model, self-regulated learning and motivation to math functioned as the most important upstream variables, mathematical self-efficacy and mathematical thinking served as the two mediating mechanisms most closely linked to performance, and broad personality traits occupied a more distal and context-sensitive explanatory position. The contribution of the study therefore lies not simply in integrating familiar variables into one model, but in clarifying how their explanatory roles differ in proximity, mechanism, and contextual salience within a mathematics-specific account of higher-order problem-solving.

### 5.1 Theoretical contributions and new knowledge

This study makes three main theoretical contributions. First, it clarifies the relative explanatory priority of distal personality traits and proximal learning processes within a single mathematics-specific framework. Although prior work has shown that both non-cognitive traits and process-related variables matter for academic outcomes (Mammadov, 2022; Cupani et al., 2025), the present findings show that motivation to math and self-regulated learning retained greater explanatory relevance than broad personality traits once the model incorporated mathematical self-efficacy and mathematical thinking. In this way, the study contributes not merely by integrating familiar constructs, but by showing how their explanatory importance changes when they are modeled together.

Second, the study refines existing accounts of mathematical problem-solving by specifying two distinct mediating mechanisms: an affective-belief pathway through mathematical self-efficacy and a cognitive pathway through mathematical thinking. Prior studies have emphasized the importance of self-efficacy for persistence and performance (Voica et al., 2020) and the role of higher-order mathematical thinking in solving complex and non-routine problems (Genç et al., 2025; Ceballos et al., 2026). The present study extends this literature by showing that these two pathways are complementary rather than interchangeable, thereby offering a more differentiated explanation of how proximal learning processes are associated with higher-order mathematical performance.

Third, the study contributes contextualized evidence from a high-performance educational system. The findings suggest that the relative salience of trait-based and process-based predictors may vary across educational settings. In the Shanghai context, variation in higher-order mathematical problem-solving was more strongly represented by self-regulated learning, motivation to math, mathematical self-efficacy, and mathematical thinking than by broad personality traits. This implies that the explanatory strength of broad personality traits may depend partly on how strongly an educational system structures and standardizes students’ observable academic behavior.

Taken together, the new knowledge provided by this study lies in showing that higher-order mathematical problem-solving is more convincingly explained, within the present sample and model, by a process-centered architecture in which self-regulated learning and motivation operate through both affective-belief and cognitive pathways, while broad personality traits occupy a more distal and context-sensitive explanatory position.

### 5.2 Practical implications

The findings of this study suggest that efforts to improve students’ mathematical problem-solving should focus less on broad assumptions about fixed aptitude or stable disposition and more on strengthening the modifiable learning processes that appear most closely associated with higher-order performance. In practical terms, this means that mathematics teaching should not be limited to transmitting procedures or increasing the amount of practice. It should also systematically support how students regulate learning, sustain motivation, build confidence, and develop the quality of their mathematical thinking.

One important implication concerns self-regulated learning. Teachers can incorporate structured opportunities for goal setting, planning, self-monitoring, error analysis, and strategic adjustment into routine mathematics instruction. In problem-solving explanation, guided practice, and homework feedback, students can be encouraged to identify what the task requires, monitor whether their current strategy is working, and revise their approach when necessary. Such practices are particularly important in high-pressure school environments, where students may complete large amounts of work without necessarily developing reflective control over how they learn.

A second implication concerns motivation to math. Teachers and schools should therefore treat mathematical motivation not as a secondary emotional outcome, but as a central instructional target. This can be supported by making mathematics more meaningful, showing its relevance to real-life and future-oriented contexts, and creating classroom climates in which sustained effort and productive struggle are valued. Opportunities for choice, peer collaboration, and multiple-solution discussion may also help students experience mathematics as something worth engaging with rather than merely something to complete under pressure.

A third implication concerns the two mechanisms most closely linked to performance: mathematical self-efficacy and mathematical thinking. In practice, this means that teachers should design learning environments that support confidence and higher-order thought at the same time. Self-efficacy can be strengthened through scaffolded challenge, process-based feedback, visible progress, and opportunities for successful engagement with demanding tasks. Mathematical thinking can be cultivated through non-routine problems, modeling activities, strategy comparison, justification-focused classroom dialog, and tasks that require students to explain not only what answer they reached, but why a solution method is valid.

The present findings also suggest a practical caution. Given the comparatively limited independent role of personality traits in the current model, schools may gain more practical leverage by investing in students’ motivation, self-regulation, confidence, and higher-order thinking development than by interpreting problem-solving differences primarily through the lens of fixed personal dispositions. This does not imply that individual differences are unimportant. Rather, it suggests that the most educationally actionable points of intervention lie in the domain-specific processes that schools and teachers can actively influence.

### 5.3 Limitations and future study

Several limitations should be noted. The data were collected at one point in time, and some key variables were measured through students’ own reports, so the findings should be interpreted with caution when discussing direction of influence. In addition, the sample came only from ordinary high schools in Shanghai, which limits the extent to which the results can be extended to other regions, school settings, or age groups.

Further research could follow students over a longer period, draw on a wider range of evidence such as teacher judgments, classroom records, or learning-process data, and test whether the same pattern appears in other educational settings. It would also be valuable to extend the present framework by bringing in other theories and variables that may help explain mathematical problem-solving more fully, such as task value, achievement emotions, teacher support, classroom climate, or math anxiety, so that the links among broad traits, proximal learning processes, and higher-order performance can be understood in a more integrated way.

## 6 Conclusion

Using a sample of Shanghai high school students, this study constructed and tested a structural model linking personality traits, motivation to math, self-regulated learning, mathematical self-efficacy, mathematical thinking, and mathematical problem-solving ability. The results indicate that, after controlling for prior mathematics achievement and multiple background variables, self-regulated learning and motivation to math showed greater relative predictive strength within the present model than broad personality traits, while mathematical self-efficacy and mathematical thinking emerged as the mechanisms most closely associated with problem-solving performance.

More specifically, the findings suggest that broad personality traits may occupy a more distal and context-sensitive explanatory position once mathematics-specific motivational, regulatory, affective, and cognitive processes are modeled simultaneously. In contrast, the strongest explanatory relevance in the current model appears to lie in the more proximal learning processes through which students regulate learning, sustain motivation, evaluate their own capability, and engage in higher-order mathematical thinking.

Overall, the study supports a process-centered interpretation of higher-order mathematical problem-solving in which students’ ways of learning, thinking, and evaluating their own competence showed greater relative explanatory relevance within the present model and this high-performance educational context than broad dispositional tendencies alone. In this sense, the study provides both empirical evidence and a theoretical framework for understanding how higher-order mathematical problem-solving may be more convincingly understood, within the present sample and model, through modifiable learning processes and their affective-belief and cognitive pathways than through broad trait-based explanations alone.

## Data Availability

The raw data supporting the conclusions of this article will be made available by the authors, without undue reservation.

## References

[ref1] Abood M. Bassam H. A. Fatin M. Ahmad M. G. (2020). The relationship between personality traits, academic self-efficacy and academic adaptation among university students in Jordan. Int. J. High. Educ. 9, 17413–17413. doi: 10.5430/ijhe.v9n3p120

[ref2] Ackerman P. L. Kanfer R. (2025). Cognitive ability and non-ability trait predictors of academic achievement: a four-year longitudinal study. J. Intelligence 13:79. doi: 10.3390/jintelligence13070079, 40710812 PMC12295058

[ref3] Adamson K. Prion S. (2013). Reliability: measuring internal consistency using Cronbach's α. Clin. Simul. Nurs. 9, e179–e180. doi: 10.1016/j.ecns.2012.12.001

[ref4] Afzaal M. Zia A. Nouri J. Fors U. (2024). Informative feedback and explainable AI-based recommendations to support students’ self-regulation. Technol. Knowl. Learn. 29, 331–354. doi: 10.1007/s10758-023-09650-0

[ref6] Arianto F. Hanif M. (2024). Evaluating metacognitive strategies and self-regulated learning to predict primary school students' self-efficacy and problem-solving skills in science learning. J. Pedagog. Res. 8, 301–319. doi: 10.33902/JPR.202428575

[ref7] Armijo I. (2025). Balanced profiles: the role of cognitive and non-cognitive competencies in Chilean higher education academic achievement. Discov. Educ. 4:302. doi: 10.1007/s44217-025-00546-y

[ref8] Asare B. Welcome N. Arthur Y. (2024). Influence of parental involvement and academic motivation on mathematical achievement: the role of students’ mathematics interest. J. Pendidik. Mat. 18, 295–312. doi: 10.22342/jpm.v18i2.pp295-312

[ref9] Aydan S. Capa-Aydin Y. (2025). What makes them self-regulated? Self-regulation procedures of academically successful students and key influences. Acta Psychol. 257:105106. doi: 10.1016/j.actpsy.2025.105106, 40412313

[ref10] Baidoo J. Ali C. (2023). Students' mathematics and real life contexts in solving algebraic word problems. Al-Jabar J. Pendidik. Mat. 14, 483–500. doi: 10.24042/ajpm.v14i2.19272

[ref11] Barana A. Boetti G. Marchisio M. (2022). Self-assessment in the development of mathematical problem-solving skills. Educ. Sci. 12:81. doi: 10.3390/educsci12020081

[ref12] Begum A. J. (2025). “Self-regulation,” in Cognitive Control Skills for Educational Success: Theory and Practice, (), 193–234.

[ref13] Bendol R. Jr R. (2025). Students’ confidence in mathematics: a comprehensive literature review. Indonesian J. Multidicipl. Res. 5, 187–196. doi: 10.17509/ijomr.v5i1.82065

[ref14] Bleidorn W. Hopwood C. J. Back M. D. Denissen J. J. A. Hennecke M. Hill P. L. . (2021). Personality trait stability and change. Pers. Sci. 2:e6009. doi: 10.5964/ps.6009

[ref15] Brenner C. A. (2022). Self-regulated learning, self-determination theory and teacher candidates’ development of competency-based teaching practices. Smart Learn. Environ. 9:3. doi: 10.1186/s40561-021-00184-5

[ref17] Ceballos H. Bogaart T. v. d. van Ginkel S. Spandaw J. Drijvers P. (2026). How collaborative problem solving promotes higher-order thinking skills: a systematic review of design features and processes. Think. Skills Creat. 59:102001. doi: 10.1016/j.tsc.2025.102001

[ref18] Chen J. (2024). “Development and validation of self-report questionnaires,” in Writing and Revising in Second Language Classrooms: The Role of Self-Regulation in Cultivating Expert Writers, (), 57–78.

[ref20] Chirove M. (2023). Secondary school learners' self-efficacy and achievement in non-routine mathematics problem-solving. Stud. Learn. Teach. 4, 508–521. doi: 10.46627/silet.v4i3.321

[ref21] Cupani M. Garrido S. J. Moran V. Ghio F. B. Azpilicueta A. E. Chemisquy S. (2025). The role of personality and learning experiences in a social–cognitive model of academic performance in mathematics. School Psychol. Int. 47:01430343251392621. doi: 10.1177/01430343251392621

[ref22] Dang T. Du W. Niu M. Xu Z. (2025). The effects of personality traits on learning engagement among college students: the mediating role of emotion regulation. Front. Psychol. 15:1476437. doi: 10.3389/fpsyg.2024.1476437, 39868019 PMC11759291

[ref23] Elagha N. Pellegrino J. W. (2024). Understanding error patterns in students' solutions to linear function problems to design learning interventions. Learn. Instr. 92:101895. doi: 10.1016/j.learninstruc.2024.101895

[ref24] Fathi J. Naderi M. Soleimani H. (2023). Professional identity and psychological capital as determinants of EFL teachers’ burnout: the mediating role of self-regulation. Porta Linguarum 2023c, 101–120. doi: 10.30827/portalin.vi2023c.29630

[ref25] Fornell C. Larcker D. F. (1981). Evaluating structural equation models with unobservable variables and measurement error. J. Mark. Res. 18, 39–50. doi: 10.2307/3151312

[ref26] Fuente J. Sander P. Garzón Umerenkova A. Urien B. Pachón-Basallo M. E O. L. (2024). The Big Five factors as differential predictors of self-regulation, achievement emotions, coping and health behavior in undergraduate students. BMC Psychol. 12:267. doi: 10.1186/s40359-024-01768-9, 38741197 PMC11092092

[ref27] Genç M. Akıncı M. Karataş İ. Çolakoğlu Ö. M. Yılmaz Tığlı N. (2025). From thinking to creativity: the interplay of mathematical thinking perceptions, mathematical communication dispositions, and creative thinking dispositions. Behav. Sci. 15:1346. doi: 10.3390/bs15101346, 41153136 PMC12561959

[ref28] George A. S. (2023). Preparing students for an AI-driven world: rethinking curriculum and pedagogy in the age of artificial intelligence. Partners Universal Innov. Res. Publ. 1, 112–136. doi: 10.5281/zenodo.10245675

[ref29] Gülşen Turgut İ. Bakır N. Ş. (2025). Different predictors of high school students' mathematics achievement. Psychol. Sch. 62, 457–474. doi: 10.1002/pits.23333

[ref30] Hadwin A. F. Rostampour R. Winne P. H. (2025). Advancing self-reports of self-regulated learning: validating new measures to assess students’ beliefs, practices, and challenges. Educ. Psychol. Rev. 37:8. doi: 10.1007/s10648-024-09977-9

[ref32] Hemmler Y. M. Ifenthaler D. (2024). Self-regulated learning strategies in continuing education: a systematic review and meta-analysis. Educ. Res. Rev. 45:100629. doi: 10.1016/j.edurev.2024.100629

[ref34] Horrocks M. Shearman D. (2025). Rethinking what is valuable in mathematics and statistics education. Int. J. Math. Educ. Sci. Technol. 56, 2513–2533. doi: 10.1080/0020739X.2025.2556864

[ref35] Huang C.-H. (2021). Using PLS-SEM model to explore the influencing factors of learning satisfaction in blended learning. Educ. Sci. 11:249. doi: 10.3390/educsci11050249

[ref36] Huang J. Cai Y. Lv Z. Huang Y. Zheng X.-L. (2024). Toward self-regulated learning: effects of different types of data-driven feedback on pupils’ mathematics word problem-solving performance. Front. Psychol. 15:1356852. doi: 10.3389/fpsyg.2024.1356852, 39411557 PMC11473304

[ref37] Jartitngarm N. (2025). Exploring self-regulated learning and motivational strategies in a flipped classroom: implications for academic achievement. LEARN J. 18, 456–492. doi: 10.70730/bzwo9910

[ref38] Jiang Y. Zhang L. (2023). High school students' expectancy, value, and cost profiles and their relations with engagement and achievement in math and English. Learn. Individ. Differ. 101:102252. doi: 10.1016/j.lindif.2022.102252

[ref39] Johansson A. Sumpter L. (2025). The role of different arguments: upper secondary school students’ collective mathematical reasoning in algebra. Int. J. Sci. Math. Educ. 23, 3153–3177. doi: 10.1007/s10763-025-10579-2

[ref40] Kamberi M. (2025). The types of intrinsic motivation as predictors of academic achievement: the mediating role of deep learning strategy. Cogent Educ. 12:2482482. doi: 10.1080/2331186X.2025.2482482

[ref41] Kappassova S. Abylkassymova A. Bulut U. Zykrina S. Zhumagulova Z. Balta N. (2025). Mathematical literacy and its influencing factors: a decade of research findings (2015-2024). Eurasia J. Math. Sci. Technol. Educ. 21:em2671. doi: 10.29333/ejmste/16615

[ref44] Kholifah N. Nurtanto M. Mutohhari F. Abi Hamid M. Mutiara I. Setiawan D. . (2025). Factors influencing student career choice in vocational education in Indonesia: a mediating effect of self-efficacy. Soc. Sci. Human. Open 11:101369. doi: 10.1016/j.ssaho.2025.101369

[ref45] Kibtiyah M. Suud F. M. (2024). Relationship between Big Five personalities and habit of memorizing the Qur'an on mathematics learning achievement through mediator of self-regulated learning. Islam. Guid. Couns. J. 7:15. doi: 10.25217/0020247453900

[ref46] Kirkham J. Chapman E. Male S. (2023). Measuring motivation for mathematics course choice in secondary school students: interrelationships between cost and other situated expectancy-value theory components. SAGE Open 13:21582440231180671. doi: 10.1177/21582440231180671

[ref47] Kock N. (2015). One-tailed or two-tailed P values in PLS-SEM? Int. J. e-Collab. 11, 1–7. doi: 10.4018/ijec.2015040101

[ref48] Kryshko O. Fleischer J. Grunschel C. Leutner D. (2022). Self-efficacy for motivational regulation and satisfaction with academic studies in STEM undergraduates: the mediating role of study motivation. Learn. Individ. Differ. 93:102096. doi: 10.1016/j.lindif.2021.102096

[ref49] Landa J. Berciano A. Marbán J. M. (2025). Moderating effect of perceived self-efficacy on university students'self-regulation in mathematics problem solving. Int. J. Sci. Math. Educ. 23, 3815–3839. doi: 10.1007/s10763-025-10597-0

[ref50] Lee J. Y. Park J. Hughes E. M. Seale K. (2025). The effects of self-regulated strategy development on mathematical writing for students with mathematics difficulties. Remedial Spec. Educ. doi: 10.1177/07419325251350708

[ref51] Lin C. (2023). “The complete structure of mathematical thinking,” in Intellectual Development and Mathematics Learning, (), 79–111.

[ref52] Liu T. (2025). Emotional stability and academic achievement among primary and secondary school students: an empirical study. Int. J. Glob. Perspect. Acad. Res. 2, 39–45. doi: 10.70339/hjw9p825

[ref53] Liu C. Bakar Z. Qianqian X. (2025). Self-regulated learning and academic achievement in higher education: a decade systematic review. Int. J. Res. Innov. Soc. Sci. IX, 4488–4504. doi: 10.47772/IJRISS.2025.90300358

[ref54] Luria E. Shalom M. Levy D. A. (2021). Cognitive neuroscience perspectives on motivation and learning: revisiting self-determination theory. Mind Brain Educ. 15, 5–17. doi: 10.1111/mbe.12275

[ref55] Mammadov S. (2022). The Big Five personality traits and academic performance: a meta-analysis. J. Pers. 90, 222–255. doi: 10.1111/jopy.12663, 34265097

[ref56] Mangarin R. Caballes D. (2024). Difficulties in learning mathematics: a systematic review. Int. J. Res. Sci. Innov. XI, 401–405. doi: 10.51244/IJRSI.2024.1109037

[ref57] Mayerhofer M. Lüftenegger M. Eichmair M. (2024). The development of mathematics expectancy-value profiles during the secondary–tertiary transition into STEM fields. Int. J. STEM Educ. 11:31. doi: 10.1186/s40594-024-00491-6

[ref58] Menlah C. K. A. Boateng F. O. (2025). Examining the effect of AI-based tutoring systems on students' mathematical problem-solving skills: the moderating role of mathematics anxiety. J. Pedagog. Sociol. Psychol. 7, 5–17. doi: 10.33902/jpsp.202536137

[ref59] Middleton J. A. Spanias P. A. (1999). Motivation for achievement in mathematics: findings, generalizations, and criticisms of the research. J. Res. Math. Educ. 30, 65–88. doi: 10.2307/749630

[ref60] Moreira P. Pedras S. Pombo P. (2020). Students’ personality contributes more to academic performance than well-being and learning approach—implications for sustainable development and education. Eur. J. Investig. Health Psychol. Educ. 10, 1132–1149. doi: 10.3390/ejihpe10040079, 34542440 PMC8314314

[ref61] Polack C. W. Miller R. R. (2022). Testing improves performance as well as assesses learning: a review of the testing effect with implications for models of learning. J. Exp. Psychol. Anim. Learn. Cogn. 48, 222–241. doi: 10.1037/xan0000323, 35446091 PMC10229024

[ref62] Prabawanto S. Herman T. Melani R. Samosir C. Mefiana S. (2024). Students’ difficulties in understanding problems and making mathematical models in the contextual problem solving process. 4th International Conference on Education and Technology (ICETECH 2023), 796–810.

[ref64] Říčan J. Chytrý V. Medová J. (2022). Aspects of self-regulated learning and their influence on the mathematics achievement of fifth graders in the context of four different proclaimed curricula. Front. Psychol. 13:963151. doi: 10.3389/fpsyg.2022.963151, 36304860 PMC9592980

[ref66] Sarwer S. Abid M. N. Chao H. Siming L. Dukhaykh S. (2025). Examining the impact of positive psychological attributes on emotional stability and academic burnout among undergraduate students: a cross-sectional study. BMC Psychol. 13:614. doi: 10.1186/s40359-025-02880-0, 40474250 PMC12142860

[ref67] Shengyao Y. Salarzadeh Jenatabadi H. Mengshi Y. Minqin C. Xuefen L. Mustafa Z. (2024). Academic resilience, self-efficacy, and motivation: the role of parenting style. Sci. Rep. 14:5571. doi: 10.1038/s41598-024-55530-7, 38448465 PMC10918079

[ref68] Shi Y. Yang H. (2025). Development, revision, and validation of a self-regulated learning questionnaire for Chinese undergraduate students. Acta Psychol. 256:104956. doi: 10.1016/j.actpsy.2025.104956, 40233651

[ref69] Shimizu Y. (2025). Learning engagement as moderator between self-efficacy, math anxiety, use of diagrams, and complex plane problem-solving. Eurasia J. Math. Sci. Technol. Educ. 21:em2586. doi: 10.29333/ejmste/15956

[ref71] Smit R. Dober H. Hess K. Bachmann P. Birri T. (2022). Supporting primary students' mathematical reasoning practice: the effects of formative feedback and the mediating role of self-efficacy. Res. Math. Educ. 25, 277–300. doi: 10.1080/14794802.2022.2062780

[ref72] Soto C. J. John O. P. (2017). The next Big Five inventory (BFI-2): developing and assessing a hierarchical model with 15 facets to enhance bandwidth, fidelity, and predictive power. J. Pers. Soc. Psychol. 113, 117–143. doi: 10.1037/pspp0000096, 27055049

[ref73] Stavropoulou G. Daniilidou A. Nerantzaki K. (2025). Exploring the interplay of motivation, self-efficacy, critical thinking, and self-regulation in predicting academic achievement among university students. F1000Res 14:344. doi: 10.12688/f1000research.161821.2, 40808874 PMC12345619

[ref74] Stenberg Hartviksen E. Haavold P. Ø. (2025). Differences between experts in mathematical problem solving. Think. Skills Creat. 58:101875. doi: 10.1016/j.tsc.2025.101875

[ref78] Sun Y. Liu L. (2023). Structural equation modeling of university students' academic resilience academic well-being, personality and educational attainment in online classes with Tencent meeting application in China: investigating the role of student engagement. BMC Psychol. 11:347. doi: 10.1186/s40359-023-01366-1, 37864215 PMC10589943

[ref79] Supriadi N. Jamaluddin Z W. Suherman S. (2024). The role of learning anxiety and mathematical reasoning as predictor of promoting learning motivation: the mediating role of mathematical problem solving. Think. Skills Creat. 52:101497. doi: 10.1016/j.tsc.2024.101497

[ref80] Suryawan I. P. P. Sudiarta I. G. P. Suharta I. G. P. (2023). Students' critical thinking skills in solving mathematical problems: systematic literature review. Indonesian J. Educ. Res. Rev. 6, 120–133. doi: 10.23887/ijerr.v6i1.56462

[ref82] Torres-Peña R. C. Peña-González D. Lara-Orozco J. L. Ariza E. A. Vergara D. (2025). Enhancing numerical thinking through problem solving: a teaching experience for third-grade mathematics. Educ. Sci. 15:667. doi: 10.3390/educsci15060667

[ref83] Treacy P. O’Meara N. Prendergast M. (2024). The role of expectancy-value theory in upper-secondary level students’ decisions to avoid the study of advanced mathematics. Ir. Educ. Stud. 43, 1175–1188. doi: 10.1080/03323315.2023.2200420

[ref84] Velez A. J. B. Abuzo E. P. (2024). Mathematics self-efficacy and motivation as predictors of problem-solving skills of students. Twist 19, 417–430. doi: 10.5281/twist.10049652#108

[ref85] Voica C. Singer F. M. Stan E. (2020). How are motivation and self-efficacy interacting in problem-solving and problem-posing? Educ. Stud. Math. 105, 487–517. doi: 10.1007/s10649-020-10005-0

[ref86] Waheed Z. Tham J. K O. (2025). The impact of self-regulated learning strategies on academic performance: a systematic review. Soc. Sci. Human Res. Bulletin 2, 398–407. doi: 10.55677/SSHRB/2025-3050-0803

[ref87] Wang T.-Y. (2025). Assessing systems thinking skills in mathematics: development and application of an operational framework. Int. J. Sci. Math. Educ. 23:3761. doi: 10.1007/s10763-025-10592-5

[ref88] Wang Y. Xie B. Zhou L. Wang L. Jin H. (2025). The influence of learning interest on complex mathematical problem-solving ability: the mediating effect of classroom disruptive behavior and self-efficacy. Front. Psychol. 16:1638695. doi: 10.3389/fpsyg.2025.1638695, 41181698 PMC12573462

[ref89] Worku D. T. Ejigu M. A. Gebremeskal T. G. Kassa Gogie T. (2025). Assessing the impact of multiple representations based instruction integrated with formative assessment practice on secondary school students’ problem-solving performance in physics. Res. Sci. Technol. Educ. 44, –249. doi: 10.1080/02635143.2025.2469062

[ref90] Xu Z. Li S. (2025). The relationship between student perceived cognitive activation strategies and mathematics academic performance: a moderated mediation model. SAGE Open 15:21582440251385866. doi: 10.1177/21582440251385866

[ref91] Xu B. Ma X. Zhang Y. Wu X. (2025). How does mathematical literacy affect creative thinking? Independent effects and differential impacts across proficiency groups. Acta Psychol. 260:105509. doi: 10.1016/j.actpsy.2025.105509, 40945153

[ref92] Yu B. (2023). Self-regulated learning: a key factor in the effectiveness of online learning for second language learners. Front. Psychol. 13:1051349. doi: 10.3389/fpsyg.2022.1051349, 36710757 PMC9877336

[ref93] Yu W. Zhou S. Zhou Y. (2023). Measuring mathematics self-efficacy: multitrait-multimethod comparison. Front. Psychol. 14:1108536. doi: 10.3389/fpsyg.2023.1108536, 36960008 PMC10028075

[ref94] Zakariya Y. F. (2022). Improving students' mathematics self-efficacy: a systematic review of intervention studies. Front. Psychol. 13:986622. doi: 10.3389/fpsyg.2022.986622, 36225698 PMC9549262

[ref95] Zakariya Y. F. Nilsen H. K. Goodchild S. Bjørkestøl K. (2022). Self-efficacy and approaches to learning mathematics among engineering students: empirical evidence for potential causal relations. Int. J. Math. Educ. Sci. Technol. 53, 827–841. doi: 10.1080/0020739X.2020.1783006

[ref96] Zarestky J. Bigler M. Brazile M. Lopes T. Bangerth W. (2022). Reflective writing supports metacognition and self-regulation in graduate computational science and engineering. Comput. Educ. Open 3:100085. doi: 10.1016/j.caeo.2022.100085

[ref97] Zhang J. Zhou Y. Jing B. Pi Z. Ma H. (2024). Metacognition and mathematical modeling skills: the mediating roles of computational thinking in high school students. J. Intelligence 12:55. doi: 10.3390/jintelligence12060055, 38921690 PMC11205218

[ref98] Zhao W. Ma R. (2025). Investigating the relationship between goal orientation, self-efficacy, positive emotionality, and affective engagement among Chinese students. Acta Psychol. 253:104735. doi: 10.1016/j.actpsy.2025.104735, 39862451

[ref100] Zimmerman B. J. (2023). “Dimensions of academic self-regulation: a conceptual framework for education,” in Self-Regulation of Learning and Performance, eds. Schunk D. H. Zimmerman B. J. (New York: Routledge), 3–21. doi: 10.4324/9780203763353-1

[ref101] Zuo S. Huang Q. Qi C. (2024). The relationship between cognitive activation and mathematics achievement: mediating roles of self-efficacy and mathematics anxiety. Curr. Psychol. 43, 30794–30805. doi: 10.1007/s12144-024-06700-3

