# Supplementary material for: Beyond personality traits: a motivation–self-regulation model of mathematical problem-solving through self-efficacy and mathematical thinking
Source: Front Psychol. 2026 Apr 13;17:1803867. doi: 10.3389/fpsyg.2026.1803867 (PMC13111107; doi:10.3389/fpsyg.2026.1803867)
Supplement: Supplementary file 1 [file Supplementary_file_1.docx]

**Appendix.**

**Appendix Table A1.Indicator Cross-Loadings of the Reflective Constructs**

| **Item** | **PT** | **MOT** | **SRL** | **MSE** | **MT** | **PS** |
| --- | --- | --- | --- | --- | --- | --- |
| PT1 | 0.71 | 0.24 | 0.27 | 0.23 | 0.2 | 0.18 |
| PT2 | 0.71 | 0.22 | 0.25 | 0.21 | 0.19 | 0.17 |
| PT3 | 0.74 | 0.23 | 0.28 | 0.22 | 0.21 | 0.18 |
| PT4 | 0.75 | 0.25 | 0.29 | 0.24 | 0.22 | 0.19 |
| PT5 | 0.76 | 0.24 | 0.3 | 0.23 | 0.22 | 0.2 |
| PT6 | 0.8 | 0.26 | 0.31 | 0.25 | 0.23 | 0.21 |
| PT7 | 0.81 | 0.27 | 0.32 | 0.26 | 0.24 | 0.22 |
| PT8 | 0.81 | 0.28 | 0.33 | 0.26 | 0.24 | 0.22 |
| PT9 | 0.83 | 0.29 | 0.34 | 0.27 | 0.25 | 0.23 |
| PT10 | 0.84 | 0.29 | 0.34 | 0.27 | 0.25 | 0.23 |
| PT11 | 0.86 | 0.3 | 0.35 | 0.28 | 0.26 | 0.24 |
| PT12 | 0.88 | 0.31 | 0.35 | 0.29 | 0.26 | 0.24 |
| MOT1 | 0.22 | 0.71 | 0.39 | 0.37 | 0.31 | 0.28 |
| MOT2 | 0.23 | 0.75 | 0.4 | 0.38 | 0.32 | 0.29 |
| MOT3 | 0.24 | 0.78 | 0.42 | 0.4 | 0.34 | 0.31 |
| MOT4 | 0.24 | 0.77 | 0.41 | 0.39 | 0.34 | 0.3 |
| MOT5 | 0.25 | 0.78 | 0.42 | 0.4 | 0.35 | 0.31 |
| MOT6 | 0.25 | 0.8 | 0.43 | 0.41 | 0.36 | 0.32 |
| MOT7 | 0.26 | 0.81 | 0.44 | 0.42 | 0.36 | 0.33 |
| MOT8 | 0.27 | 0.85 | 0.46 | 0.44 | 0.38 | 0.35 |
| MOT9 | 0.27 | 0.85 | 0.46 | 0.44 | 0.38 | 0.35 |
| MOT10 | 0.28 | 0.88 | 0.47 | 0.45 | 0.39 | 0.36 |
| SRL1 | 0.28 | 0.39 | 0.75 | 0.36 | 0.33 | 0.28 |
| SRL2 | 0.28 | 0.39 | 0.74 | 0.35 | 0.32 | 0.28 |
| SRL3 | 0.29 | 0.4 | 0.76 | 0.36 | 0.33 | 0.29 |
| SRL4 | 0.29 | 0.41 | 0.78 | 0.37 | 0.34 | 0.29 |
| SRL5 | 0.3 | 0.41 | 0.78 | 0.37 | 0.34 | 0.3 |
| SRL6 | 0.31 | 0.42 | 0.82 | 0.39 | 0.36 | 0.31 |
| SRL7 | 0.31 | 0.43 | 0.81 | 0.39 | 0.36 | 0.31 |
| SRL8 | 0.32 | 0.44 | 0.84 | 0.4 | 0.37 | 0.32 |
| SRL9 | 0.33 | 0.45 | 0.85 | 0.41 | 0.38 | 0.33 |
| SRL10 | 0.33 | 0.45 | 0.86 | 0.42 | 0.38 | 0.34 |
| SRL11 | 0.34 | 0.46 | 0.88 | 0.43 | 0.39 | 0.34 |
| MSE1 | 0.22 | 0.37 | 0.35 | 0.7 | 0.41 | 0.31 |
| MSE2 | 0.23 | 0.38 | 0.36 | 0.73 | 0.42 | 0.32 |
| MSE3 | 0.24 | 0.39 | 0.37 | 0.76 | 0.43 | 0.33 |
| MSE4 | 0.25 | 0.4 | 0.38 | 0.8 | 0.45 | 0.35 |
| MSE5 | 0.25 | 0.4 | 0.38 | 0.79 | 0.45 | 0.35 |
| MSE6 | 0.26 | 0.42 | 0.39 | 0.84 | 0.47 | 0.37 |
| MSE7 | 0.27 | 0.43 | 0.4 | 0.86 | 0.48 | 0.38 |
| MSE8 | 0.28 | 0.44 | 0.41 | 0.88 | 0.49 | 0.39 |
| MT1 | 0.2 | 0.31 | 0.32 | 0.41 | 0.73 | 0.46 |
| MT2 | 0.21 | 0.32 | 0.33 | 0.42 | 0.76 | 0.48 |
| MT3 | 0.21 | 0.33 | 0.34 | 0.43 | 0.77 | 0.49 |
| MT4 | 0.22 | 0.34 | 0.35 | 0.44 | 0.79 | 0.5 |
| MT5 | 0.23 | 0.35 | 0.36 | 0.46 | 0.82 | 0.52 |
| MT6 | 0.24 | 0.36 | 0.37 | 0.47 | 0.83 | 0.53 |
| MT7 | 0.25 | 0.37 | 0.38 | 0.48 | 0.86 | 0.55 |
| MT8 | 0.26 | 0.38 | 0.39 | 0.49 | 0.88 | 0.56 |
| PS1 | 0.18 | 0.27 | 0.26 | 0.31 | 0.46 | 0.75 |
| PS2 | 0.18 | 0.28 | 0.27 | 0.32 | 0.47 | 0.75 |
| PS3 | 0.19 | 0.29 | 0.28 | 0.33 | 0.48 | 0.77 |
| PS4 | 0.2 | 0.3 | 0.29 | 0.35 | 0.5 | 0.81 |
| PS5 | 0.21 | 0.31 | 0.3 | 0.36 | 0.52 | 0.84 |
| PS6 | 0.22 | 0.32 | 0.31 | 0.37 | 0.54 | 0.88 |

Appendix Table A2. Full Measurement Items, Adaptation Basis, and Response Format

| **Construct** | **Item code** | **Full item wording** | **Response format(**1 = strongly disagree to 5 = strongly agree**)** |
| --- | --- | --- | --- |
| Personality Traits (PT) | PT1 | I usually complete my mathematics study tasks on time. |  |
|  | PT2 | I try to stay organized when learning mathematics. |  |
|  | PT3 | I keep working on mathematics tasks even when they are difficult. |  |
|  | PT4 | I pay close attention to details in mathematics learning. |  |
|  | PT5 | I remain calm when I face difficult mathematics problems. |  |
|  | PT6 | I can manage my frustration when I do not solve a mathematics problem immediately. |  |
|  | PT7 | I do not easily lose confidence during mathematics learning. |  |
|  | PT8 | I can stay emotionally steady during mathematics tests or challenging tasks. |  |
|  | PT9 | I am interested in exploring different ways to solve a mathematics problem. |  |
|  | PT10 | I enjoy learning new mathematical ideas and methods. |  |
|  | PT11 | I like thinking about unfamiliar or complex mathematics questions. |  |
|  | PT12 | I am willing to try new approaches in mathematics learning. |  |
| Motivation to Math (MOT) | MOT1 | I enjoy learning mathematics. |  |
|  | MOT2 | Mathematics is one of the subjects that interests me most. |  |
|  | MOT3 | I think learning mathematics is important for my future development. |  |
|  | MOT4 | Mathematics is useful in real life. |  |
|  | MOT5 | I want to understand mathematics deeply, not just remember formulas. |  |
|  | MOT6 | I am willing to spend extra time improving my mathematics learning. |  |
|  | MOT7 | I try to do well in mathematics because I value the subject. |  |
|  | MOT8 | I feel motivated when I am challenged by mathematics tasks. |  |
|  | MOT9 | I want to master difficult mathematics topics step by step. |  |
|  | MOT10 | Even when mathematics is hard, I still want to keep learning it. |  |
| Self-Regulated Learning in Mathematics (SRL) | SRL1 | Before studying mathematics, I set clear learning goals. |  |
|  | SRL2 | I make a plan for how to complete my mathematics study tasks. |  |
|  | SRL3 | I decide in advance how much time I need for mathematics study. |  |
|  | SRL4 | While solving mathematics problems, I check whether I really understand what I am doing. |  |
|  | SRL5 | I pay attention to whether my solution method is working. |  |
|  | SRL6 | I notice when I am confused in mathematics learning. |  |
|  | SRL7 | I ask myself whether my answer makes sense. |  |
|  | SRL8 | When one method does not work, I try another way to solve the mathematics problem. |  |
|  | SRL9 | I adjust my study strategies when I find them ineffective. |  |
|  | SRL10 | I review my mistakes in mathematics to improve my later performance. |  |
|  | SRL11 | I change my effort or strategy when I meet difficulties in mathematics learning. |  |
| Math Self-Efficacy (MSE) | MSE1 | I am confident that I can understand difficult mathematics content. |  |
|  | MSE2 | I believe I can solve challenging mathematics problems. |  |
|  | MSE3 | I am confident that I can learn new mathematics topics well. |  |
|  | MSE4 | I can do well in mathematics if I put in the necessary effort. |  |
|  | MSE5 | I believe I can find a way to solve unfamiliar mathematics problems. |  |
|  | MSE6 | I can stay effective when solving complex mathematics tasks. |  |
|  | MSE7 | I am confident that I can perform well on mathematics tests. |  |
|  | MSE8 | Even when mathematics is difficult, I believe I can succeed. |  |

Appendix Table A3. Summary of Analytic Rubric Criteria and Scoring Procedures for the Performance-Based Tasks

| **Construct** | **Task type** | **Scoring dimension** | **Score range** | **Descriptor summary** |
| --- | --- | --- | --- | --- |
| Mathematical Thinking (MT) | Non-routine/application item | Reasoning quality | 0–4 | 0 = no meaningful reasoning or blank response; 1 = minimal, fragmented, or weakly relevant reasoning; 2 = partially correct reasoning with important gaps; 3 = mostly correct and logically organized reasoning with minor weaknesses; 4 = clear, coherent, and mathematically sound reasoning. |
| Mathematical Thinking (MT) | Non-routine/application item | Modeling/generalization | 0–4 | 0 = no meaningful representation, abstraction, or generalization; 1 = very limited attempt at representation or pattern identification; 2 = partially appropriate representation or emerging generalization; 3 = appropriate modeling or generalization with minor limitations; 4 = accurate abstraction, pattern recognition, or generalization fully aligned with the problem requirements. |
| Mathematical Thinking (MT) | Non-routine/application item | Justification clarity | 0–4 | 0 = no justification; 1 = unclear, weakly connected, or largely unsupported justification; 2 = partially relevant justification with limited coherence; 3 = relevant and mostly clear justification; 4 = precise, logically connected, and mathematically well-supported justification. |
| Mathematical Problem-Solving Skills (PS) | Contextual word-problem item | Problem interpretation | 0–4 | 0 = misinterprets the problem or provides no meaningful attempt; 1 = identifies limited information but misunderstands key conditions; 2 = partial understanding of the problem situation; 3 = mostly accurate interpretation with minor omissions; 4 = accurate and complete interpretation of the contextualized problem. |
| Mathematical Problem-Solving Skills (PS) | Contextual word-problem item | Strategy selection and execution | 0–4 | 0 = no appropriate strategy; 1 = inappropriate, incomplete, or poorly matched strategy; 2 = partially appropriate strategy with major execution flaws; 3 = generally appropriate strategy with minor execution errors; 4 = appropriate, efficient, and logically executed strategy. |
| Mathematical Problem-Solving Skills (PS) | Contextual word-problem item | Solution accuracy and explanation | 0–4 | 0 = incorrect or missing solution; 1 = minimally developed response with substantial inaccuracy; 2 = partially correct solution or incomplete explanation; 3 = mostly correct solution with limited explanation weaknesses; 4 = correct solution with clear and sufficient explanation. |

**Scoring procedures.** Student responses to the mathematical thinking and mathematical problem-solving tasks were scored using analytic rubrics developed in line with the theoretical definitions of the two constructs and informed by recent frameworks for mathematical literacy, reasoning, and contextual problem solving. Each task was scored at the item level using common score anchors, and responses were evaluated dimension by dimension rather than holistically so that qualitative differences in reasoning, modeling, strategy use, interpretation, and justification could be captured more transparently.

For mathematical thinking tasks, scoring focused primarily on the quality of students’ reasoning, abstraction/modeling, and justification. For mathematical problem-solving tasks, scoring focused primarily on students’ interpretation of the problem situation, appropriateness of strategy selection and execution, and accuracy and clarity of the final solution. Higher scores consistently indicated stronger evidence of the targeted latent capability.

All responses were scored by trained raters with backgrounds in mathematics education. Before formal scoring, the raters participated in a calibration session using anchor responses to develop a shared interpretation of the rubric criteria and score levels. During formal scoring, responses were scored independently according to the scoring guide. Any disagreements were discussed and reconciled through consensus-based review; when necessary, the final score was determined after an additional review of the response against the rubric descriptors. The resulting item-level scores were aggregated within each task set to generate the total observed scores used as indicators of Mathematical Thinking and Mathematical Problem-Solving Skills in the structural model.

**Note.** The rubric was designed not merely to classify answers as correct or incorrect, but to capture the quality of students’ higher-order mathematical performance, including their reasoning process, representation or modeling ability, strategy use, and justification. This scoring logic is consistent with the study’s conceptual distinction between mathematical thinking and mathematical problem-solving as related but non-identical higher-order constructs.
